# Mechanism of Growth Regulation of Yeast Involving Hydrogen Sulfide From *S*-Propargyl-Cysteine Catalyzed by Cystathionine-γ-Lyase

**DOI:** 10.3389/fmicb.2021.679563

**Published:** 2021-07-02

**Authors:** Zhongkai Gu, Yufan Sun, Feizhen Wu, Xiaomo Wu

**Affiliations:** ^1^The Institute of Biomedical Sciences, Fudan University, Shanghai, China; ^2^Key Laboratory of Medical Molecular Virology of Ministries of Education and Health, Department of Medical Microbiology, School of Basic Medical Sciences, Fudan University, Shanghai, China; ^3^Dermatology Institute of Fuzhou, Dermatology Hospital of Fuzhou, Fuzhou, China

**Keywords:** hydrogen sulfide, H_2_S metabolism, fungal growth rate, SPRC, cystathionine-γ-lyase, fungal growth

## Abstract

Pathogenic fungi are recognized as a progressive threat to humans, particularly those with the immunocompromised condition. The growth of fungi is controlled by several factors, one of which is signaling molecules, such as hydrogen sulfide (H_2_S), which was traditionally regarded as a toxic gas without physiological function. However, recent studies have revealed that H_2_S is produced enzymatically and endogenously in several species, where it serves as a gaseous signaling molecule performing a variety of critical biological functions. However, the influence of this endogenous H_2_S on the biological activities occurring within the pathogenic fungi, such as transcriptomic and phenotypic alternations, has not been elucidated so far. Therefore, the present study was aimed to decipher this concern by utilizing *S*-propargyl-cysteine (SPRC) as a novel and stable donor of H_2_S and *Saccharomyces cerevisiae* as a fungal model. The results revealed that the yeast could produce H_2_S by catabolizing SPRC, which facilitated the growth of the yeast cells. This implies that the additional intracellularly generated H_2_S is generated primarily from the enhanced sulfur-amino-acid-biosynthesis pathways and serves to increase the growth rate of the yeast, and presumably the growth of the other fungi as well. In addition, by deciphering the implicated pathways and analyzing the *in vitro* enzymatic activities, cystathionine-γ-lyase (*CYS3*) was identified as the enzyme responsible for catabolizing SPRC into H_2_S in the yeast, which suggested that cystathionine-γ-lyase might play a significant role in the regulation of H_2_S-related transcriptomic and phenotypic alterations occurring in yeast. These findings provide important information regarding the mechanism underlying the influence of the gaseous signaling molecules such as H_2_S on fungal growth. In addition, the findings provide a better insight to the *in vivo* metabolism of H_2_S-related drugs, which would be useful for the future development of anti-fungal drugs.

## Introduction

Many fungi are recognized as opportunistic pathogens, causing invasive infections and critical illness in humans (Romani, 2011; Köhler et al., 2017). The growth of fungi is subject to several factors, one of which is signaling molecules, including hydrogen sulfide (H_2_S), which plays a central role in intercellular communication and intracellular redox balancing in yeast, a member of kingdom fungi (Lloyd, 2006). H_2_S ranks the third as a gaseous signal molecule after nitric oxide (NO) and carbon monoxide (CO). H_2_S is reported to be canonically involved in regulating a variety of physiological functions in mammals (Abe and Kimura, 1996; Hosoki et al., 1997; Kimura and Kimura, 2004; Li et al., 2005; Luan et al., 2012; Olas, 2015; Yu et al., 2014). However, despite the promising results observed with the use of H_2_S in animal models, the influence of endogenously-generated H_2_S on the transcriptomic and phenotypic alternations in fungi has been largely overlooked. Moreover, the canonical donors of exogenous H_2_S, such as sulfide salts, are reported to cause a spike in the generation of H_2_S, resulting in an unstable level of H_2_S concentration (Li et al., 2009; Zhao et al., 2013). *S*-propargyl-cysteine (SPRC) was developed to replace sulfide salts for a gentle and stable generation of H_2_S (Wang et al., 2009). In this context, the present study employed SPRC as the donor of H_2_S and budding yeast *Saccharomyces cerevisiae* as the model organism to investigate the influence of H_2_S on the transcriptome and phenotype alterations in the yeast to identify any potential H_2_S-related factor for inhibition by drugs (Denoth Lippuner et al., 2014; Mulla et al., 2014; Voisset and Blondel, 2014).

The existing literature on endogenous H_2_S in yeast mainly concerns the mechanism principally regarding the enhanced generation of H_2_S during yeast fermentation and the strategies and approaches for reducing the H_2_S production in the wine industry (Huang et al., 2017; Huo et al., 2018). The phenotypical alterations occurring in yeast due to the generation of H_2_S have not been thoroughly studied so far. In addition, the transcriptomic variations in yeast upon H_2_S production from an H_2_S donor also require further exploration and investigation.

Interestingly, the results of the present study revealed that H_2_S is intracellularly generated during the metabolism of SPRC and it can significantly enhance the growth of the yeast cells compared to NaHS. This phenomenon was initially inferred as a possibility that the growth of yeast and even that of other fungi could be inhibited by suppressing the generation of H_2_S. Moreover, the transcriptome analysis and the functional assays elucidated the molecular mechanism underlying the H_2_S-mediated growth enhancement, which further revealed that H_2_S-mediated upregulation of certain genes resulted in the enrichment of the sulfur-amino-acid biosynthesis pathways. Consequently, a pathway analysis was conducted, which identified cystathionine-γ-lyase (*CYS3*) as the catabolic enzyme candidate that could utilize SPRC as a substrate for the production of H_2_S with the enhancement the growth rate of yeast, and a further confirmation of this was provided by *in vitro* catalysis assay. Furthermore, the evolutionary analysis revealed that cystathionine-γ-lyase is highly conserved among various species of fungi (Krück et al., 2009), suggesting that Cys3p could be worth of consideration for anti-fungal drug development to inhibit fungal growth in the future.

## Materials and Methods

### Yeast Culture Conditions

The budding yeast *S. cerevisiae* strain S288C was cultured in the SD medium (Yeast Protocols Handbook, Clontech, Protocol No.: PT3024–1, Version No.: PR973283). SPRC was provided by Prof. Yizhun Zhu. Sodium hydrosulfide (NaHS) was a domestic product of Sinopharm (Shanghai, China). The S288C strain was inoculated in each well of a 96-well plate at the starting concentration of 1.7 × 10^5^ cells/mL in 150 μL medium, and the plate was subsequently incubated at 30°C in a Higro 220V incubator (DigiLab, Marlborough, MA, United States) at 600 rpm. The medium in each well contained SPRC (0.1–10 mM) or NaHS (1–8 μM) or no drug, and each well had a triplication. Every 2 h, OD_595 nm_ measurements were performed by placing the plate in a DTX880 plate reader (Beckman Coulter, Brea, CA, United States) after being subjected to shaking at 950 rpm for 1 min in the shaking module of an automation workstation (Beckman Coulter, Brea, CA, United States) (Supplementary Tables 1, 2). The experiment was performed for a minimum of 24 h. Besides, another set of yeast cells was simultaneously growing in SD medium without any drug for 30 h for the consequent standard curve measurement. A hemocytometer was applied to the measurement, and the cell density in the 1× medium suspension was known as 2.75 × 10^8^. Subsequently, a series dilution was performed by accurate pipetting from 0.8 to 0.2× and the theoretical cell densities of the diluted suspensions were known by calculation. Following, all the suspension samples, including a blank SD medium sample, were injected to 3 wells of a 96-well plate for a triplication, and each well contained a volume of 150 μL, which was followed by the OD_595 nm_ measurement (Supplementary Table 3). All the OD values together with the calculated densities were carried out for a linear regression calculation to obtain the standard curve (Supplementary Figure 1), which converted each OD value above to a corresponding cell density number.

### Transcriptome Analysis

The budding yeast cells were cultured for 16 h in a medium containing 2 mM SPRC, after which the cells were collected through centrifugation, fixed in 75% ethanol, and finally maintained at 4°C until the subsequent RNA extraction procedure. The total-RNA samples were extracted using the Biomiga Yeast RNA kit (Cat. No.: R6617, San Diego, CA, United States). The RNA-Seq library constructions were performed on the RNA samples by an RNA library prep kit (Illumina, San Diego, CA, United States Cat. No.: RS-122-2001). The library samples were sequenced by an Illumina HiSeq 2500 sequencer (San Diego, CA, United States). The raw data were consequently examined for the sequencing quality by the FastQC v0.11.9^1^, and all the reads were trimmed by the Trim_galore v0.6.6^2^ with the default setting for a better quality. The trimmed data was aligned against the *S. cerevisiae* reference genome and annotation (R64-1-1^3^) by the software Tophat v2.1.1 (Trapnell et al., 2012), and the generated FPKM data was transferred into the TPM format by TPMCalculator (Vera Alvarez et al., 2018), which was used for the generation of the enrichment heatmap by Clustvis (Metsalu and Vilo, 2015). Meanwhile, the Tophat-generated data was processed using Cuffdiff (Trapnell et al., 2012) to generate the data of gene expression differences, which was utilized to generate the volcano plot using the Origin 2018 software (OriginLab Co., Northampton, MA, United States). Subsequently, certain genes with *p*-values below 0.05 and the values of log_2_(fold change) ≥ 0.585 or ≤−0.585 were selected from the matrix for the following analyses (Supplementary Table 5). The gene ontology (GO) and KEGG analyses were performed using DAVID (Huang da et al., 2009a, b). The GSEA analysis was performed using the GSEA software (Mootha et al., 2003; Subramanian et al., 2005). The enrichment gene ontology (GO) network was deciphered using Metascape.org (Tripathi et al., 2015), and the network file was processed and visualized in Cytoscape (Shannon et al., 2003).

### Cystathionine-γ-Lyase Cloning, Expression and Purification

The gDNA of the S288C strain was extracted from the yeast cells and purified using a gDNA kit (Biomiga, San Diego, CA, United States). Subsequently, the open reading frame of the yeast cystathionine-γ-lyase *CYS3* gene was amplified by performing a primary PCR reaction with the gDNA of the S288C strain using the Q5 HF DNA polymerase (New England BioLabs, Cat. No.: M0491, Ipswich, MA, United States), the forward primer 5′-ATGACTCTACAAGAATCTGA-3′, and the reverse primer 5′-TTAGTTGGTGGCTTGTTTCA-3′. The primary PCR reaction conditions were: initial denaturation at 98°C for 30 s, followed by 35 cycles of 98°C for 10 s, 55°C for 30 s, and 72°C for 75 s, and then a final extension at 72°C for 10 min followed by reservation 4°C. The resultant PCR product was analyzed using agarose gel electrophoresis. Next, the restriction enzyme sites for *Bam*HI and *Xho*I were introduced to the ends of these amplified fragments in a secondary PCR reaction conducted using KOD Plus polymerase (TOYOBO, Japan), the forward primer 5′-CGGGATCCATGACTC TACAAGAATCTGATAAATTTG-3′, and the reverse primer 5′-CCGCTCGAGTTAGTTGGTGGCTTGTTTCAAG-3′. The secondary PCR reaction conditions were as follows: initial denaturation at 94°C for 5 min, followed by 30 cycles of 94°C for 30 s, 57°C for 30 s, and 68°C for 90 s, and then a final extension at 68°C for 10 min and reservation at 4°C. The amplified fragments from the secondary PCR reaction and an *Escherichia coli* expression vector, which was a modified pET-28a plasmid vector with a His_6_-tag and a SUMO-tag (Cheng et al., 2015), were individually digested by *Bam*HI and *Xho*I restriction enzymes (New England Biolabs, Ipswich, MA, United States), and all the digested products were examined together on agarose gel. Next, the digested fragments and the vector plasmid were ligated by overnight incubation with T4 DNA ligase (Ipswich, MA, United States) at 16°C. The constructs were transformed into Top10 competent *E. coli* and then placed onto LB agar plates containing kanamycin. A single colony was transferred to the liquid LB medium containing kanamycin for plasmid amplification and then purified using a plasmid kit (Qiagen, Hilden, Germany). After confirmation of the plasmid in the sequencing test conducted with T7 primers, the plasmid was transformed into another competent *E. coli* strain, BL21 (DE3), and then placed onto LB agarose plates containing kanamycin. On the next day, a single colony was selected for inoculation in the liquid LB medium containing kanamycin. Massive cell culture was subsequently prepared in Erlenmeyer flasks of 1 L capacity. After 18 h of culturing, when the medium OD_600_ was over 0.4, protein expression was induced by adding 0.1 mM IPTG to the culture (Isopropyl β-D-1-thiogalactopyranoside), followed by incubation at 37°C for 3 h.

The cells were harvested through centrifugation, resuspended in lysis buffer [40 mM Tris-HCl pH = 8.0, 300 mM NaCl, 6 mM MgCl_2_, 1 mM β-mercaptoethanol, 10 μM pyridoxal-phosphate, and 10 mg/mL DNase], and disrupted using a high atmospheric compressor (AH-100B, ATS Engineering Ltd., Suzhou, China) at 1,500 bar. The cell debris was removed through centrifugation at 15,000 rpm for 35 min. The proteins suspended in the supernatant were examined by SDS-PAGE, and the lysate was subsequently loaded onto a nickel column (Thermo Fisher, Waltham, MA, United States). Next, the SUMO-tag was digested by overnight incubation with SUMO proteinase (or ULP1, Thermo Fisher, Waltham, MA, United States) at 4°C, and then the elution buffer [20 mM Tris-HCl pH = 8.0, 150 mM NaCl, 6 mM MgCl_2_, and 1 mM β-mercaptoethanol] was loaded onto the column for elution. The eluted protein was diluted to 12.8% (v/v) using the dilution buffer [40 mM Tris-HCl pH = 8.0, 6 mM MgCl_2_, and 1 mM β-mercaptoethanol] and then applied to an ÄKTApurifier UPC-100 purification system (GE Healthcare, Chicago, IL, United States) equipped with a Source Q column (GE Healthcare, Chicago, IL, United States) at 4°C, using Washing Buffer A [20 mM Tris-HCl pH = 8.0, 6 mM MgCl_2_, and 1 mM β-mercaptoethanol], Washing Buffer B [20 mM Tris-HCl pH = 8.0, 1 M NaCl, 6 mM MgCl_2_, and 1 mM β-mercaptoethanol], and an interception tube (Merck-Millipore, Burlington, MA, United States). Next, the concentrated solution was centrifuged at 13,000 rpm and 4°C for 10 min, and the resultant supernatant was loaded onto a SuperdexTM 200 column (GE Healthcare, Chicago, IL, United States) that had been previously installed onto the ÄKTApurifier UPC-100 purification system, followed by washing with the washing buffer [40 mM HEPES pH = 7.5, 100 mM NaCl, 2 mM MgCl_2_, and 1 mM β-mercaptoethanol] at the rate of 0.5 mL/min. The samples were examined using SDS-PAGE and then stored at −80°C. The identity of the protein sample was confirmed using a mass spectrometer (LTQ Orbitrap XL, Thermo Fisher, Waltham, MA, United States).

### *In vitro* Catalytic Functional Assay

The purified cystathionine-γ-lyase protein (Cys3p) solution was measured for A_280_ by an Eppendorf BioSpectrometer^®^ (Eppendorf, Hamburg, Germany Cat.: 6135000009) and the value was 2.3. Meanwhile, the Abs 0.1% of Cys3p was known of 0.611 on ExPaSy (Artimo et al., 2012). By these values, the concentration of the purified protein was identified as 89 μM. Consequently, for the catalysis assay, the purified Cys3p solution was diluted to 5 μM and incubated with 500 μM of SPRC or cystathionine at 37°C on a shaking incubator (ThermoMixer^®^ C, Eppendorf, Hamburg, Germany Cat.: 5382000023) for the duration from 0 to 24 h. On the contrary, for the control assay of the inhibition of Cys3p, 500 μM of PAG (propargylglycine) was added into the mixture of 5 μM Cys3p and 500 μM SPRC, which served as the specific inhibitor to Cys3p. At each time point, a small volume was aliquoted from each sample and stored immediately at −20°C. The LC-MS/MS analysis methods for detecting SPRC, H_2_S, and cystathionine described in a previous report were used (Tan et al., 2017).

### Evolutionary Analysis of the Cystathionine-γ-Lyase of Multiple Species

The protein sequences of the cystathionine-γ-lyase of 12 species were retrieved from the protein database of NCBI (National Center of Biotechnology Information). The multiple alignment analysis was performed using COBALT (Constraint-based Multiple Alignment Tool) (Papadopoulos and Agarwala, 2007) on NCBI with default parameter settings. The consensus motifs were analyzed using ClustalΩ (Sievers et al., 2011) on EMBL (Li et al., 2015) with default parameter settings, and the generated result file from EMBL-ClustalΩ was opened and visualized in Jalview (Waterhouse et al., 2009). A phylogenetic tree in rooted format was also generated simultaneously from the EMBL-ClustalΩ using default parameter settings and subsequently visualized in Dendroscope (Huson et al., 2007; Huson and Scornavacca, 2012; Huson and Linz, 2018).

## Results

### Emission of Intracellular H_2_S Generated From Catabolized SPRC in Yeast

**FIGURE 1:**
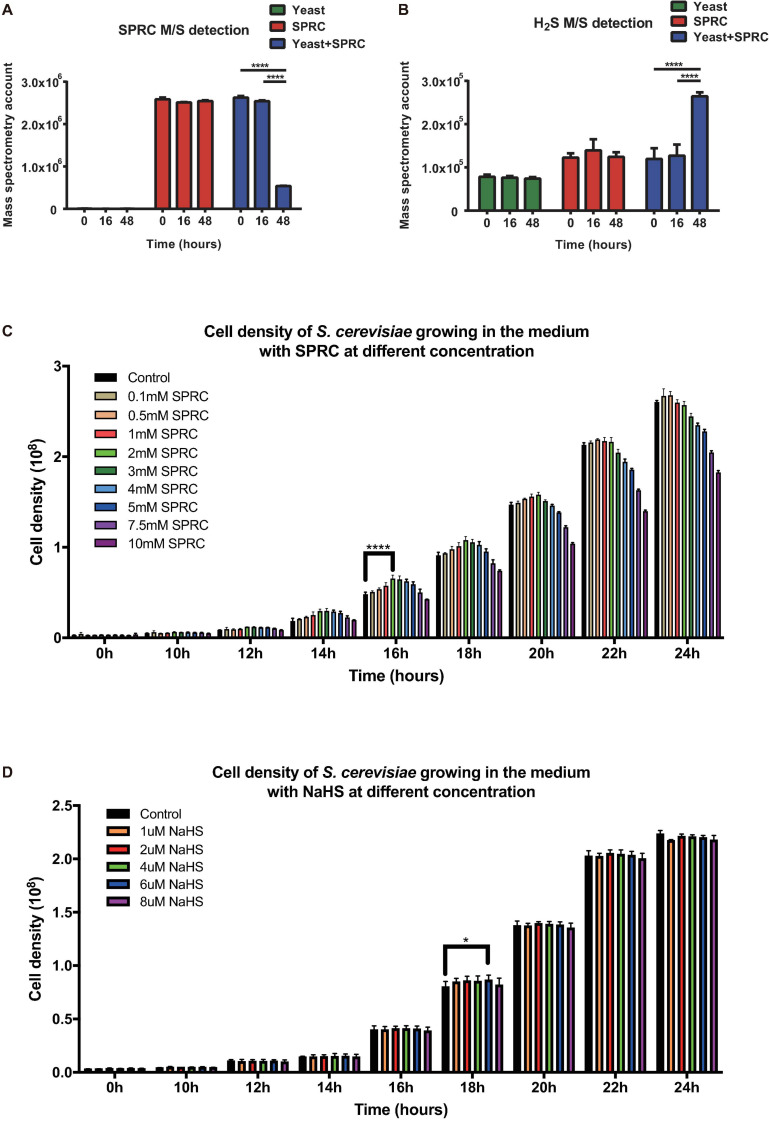
Yeast capable of catalyzing SPRC (*S*-propargyl-cysteine) and emitting H_2_S. **(A)** The mass spectrometry detection of SPRC shows the decreasing SPRC M/S signals when metabolized by the yeast cells. The horizontal axis shows the time group. The vertical axis shows the M/S signal counts. The data of triplicates was represented as the mean ± SD (Two-way ANOVA, *p*-values are of the 0 h group and 18 h in comparison to the 48 h group individually, ****p ≤ 0.0001). The green bars show the SPRC signals in the supernatant SD medium when yeast cells were cultivated. The red bars show the SPRC signals in the supernatant SD medium without any yeast cells. The blue bars show the SPRC signals in the supernatant SD medium containing SPRC when yeast cells were cultivated. **(B)** The mass spectrometry detection of H_2_S showed the increasing H_2_S M/S signals when SPRC is metabolized by the yeast cells. The horizontal axis shows the time group. The vertical axis shows the M/S signal counts. The data of triplicates was represented as the mean ± SD (Two-way ANOVA, *p*-values are of the 0 h group and 18 h in comparison to the 48 h group individually, ****p ≤ 0.0001). The green bars show the H_2_S signals in the supernatant SD medium when yeast cells were cultivated. The red bars show the H_2_S signals in the supernatant SD medium without any yeast cells. The blue bars show the H_2_S signals in the supernatant SD medium containing SPRC when yeast cells were cultivated. **(C)** The growth rate of the yeast cells at 0–24 h with the 0.1–3 mM SPRC (Two-way ANOVA, *p*-values are of the 0.5–3.0 mM SPRC group in comparison to the 0.1 mM SPRC group individually, ****p ≤ 0.0001). **(D)** The growth rate of the yeast cells at 0–24 h with the 1–8 μM NaHS (Two-way ANOVA, *p*-values are of the 2–8 μM NaHS group in comparison to the 1 μM NaHS group individually, *p ≤ 0.1).

In order to elucidate the influence of H_2_S on fungi, the S288C strain of budding yeast *S. cerevisiae* was employed as the model organism first to examine if H_2_S could be generated during the catabolization of SPRC by the yeast and then to explore if any transcriptomic and phenotypic alterations occurred in the yeast when H_2_S was served. After inoculation, the yeast cells were cultivated in the presence or absence of 2 mM SPRC for 16–48 h before harvest. After centrifugation of the harvested culture, the supernatant samples were collected and subjected to the mass-spectrometry measurement to detect SPRC and H_2_S. As depicted in Figure 1A, the SPRC signal did not change in the yeast culture for 16 h after the inoculation, following which a substantial decrease was observed in the next 32 h with an 80% decline in the signal. The control group showed no such decrease, suggesting that SPRC was catabolized by the yeast cells, presumably during the exponential growth phase. Meanwhile, the signal of H_2_S increased significantly during the same period (Figure 1B) in the SPRC-serving yeast cells, suggesting that the additionally elevated H_2_S concentrations were linked with SPRC, which was the only extracellular source of sulfur, thereby confirming that the extra H_2_S was derived intracellularly from the catabolism of SPRC within the yeast cells.

### Yeast Growth Enhancement With SPRC-Generated H_2_S

Although the results above have confirmed that H_2_S is generated via SPRC catabolism, no previous studies have reported any potential phenotype alteration in the yeast upon the generation of H_2_S. Therefore, in the present study, the growth curves of the yeast cells were measured in the presence or absence of SPRC. As depicted in Figure 1C, comparing to the control condition, the presence of SPRC enhanced the growth rate of yeast with the generation of H_2_S, particularly at the working concentrations above 0.5 mM. This growth-enhancing effect was largely dose-dependent, with 2 mM of SPRC being the most effective concentration presenting the highest growth. Interestingly, when the concentration of SPRC was above 2 mM, the enhancement of growth was undermined. The growth enhancement began to appear at the time point of around 10 h, with the highest effect observed at 16 h, which lasted for approximately 10 h. In comparison, supplementation with NaHS, a sulfide salt with rapid and uncontrolled reactivity, exhibited only a minor growth-stimulation effect on the yeast cells (Figure 1D). Therefore, it was inferred that, as a catabolic product of SPRC, the generated H_2_S was found accompanying the enhancement of the growth rate of yeast as a phenotypic alternation, while the exogenously-supplied H_2_S could not achieve this effect.

### SPRC-Generated H_2_S Led to Elevated Expression of Genes Involved in Sulfur–Amino Acid Biosynthesis

**FIGURE 2:**
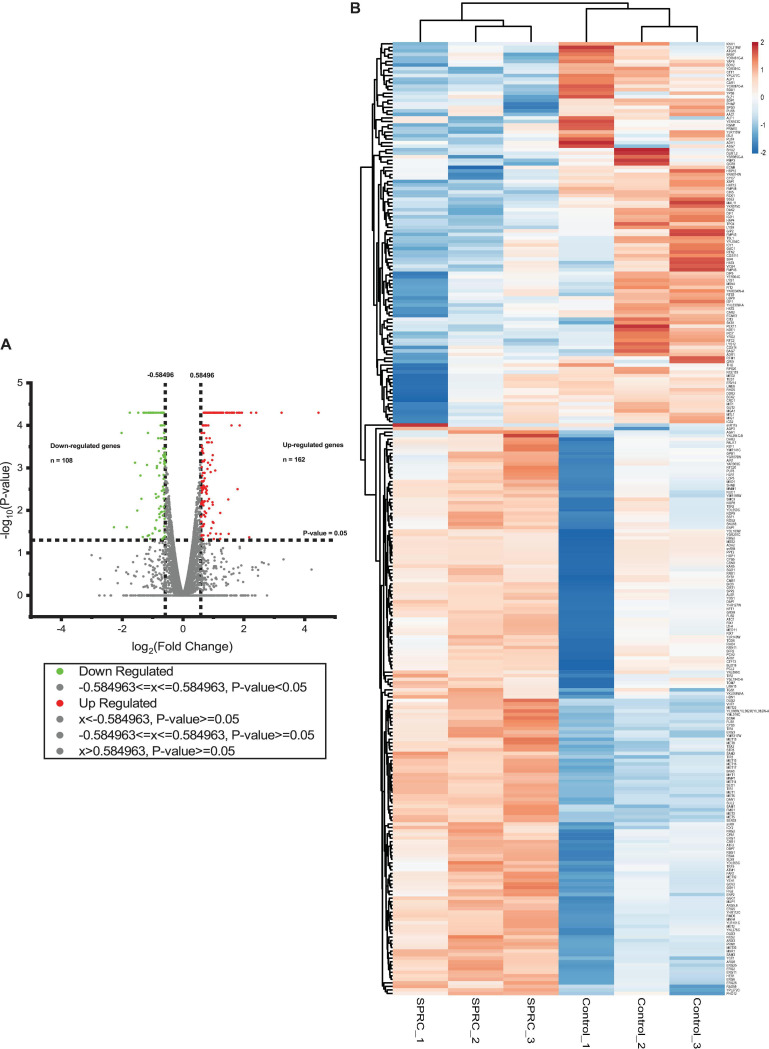
The volcano plot and the heatmap of differentially expressed genes in response to SPRC display that SPRC-upregulated genes are more than SPRC-downregulated genes. **(A)** The volcano plot represents the differentially expressed genes under the SPRC treatment comparing with the control condition. The horizontal axis shows the value of log_2_(fold change) of each gene which is represented by a spot, and the cut-off values are set to ±0.584963. The vertical axis shows the –log_10_(*p*-value) of each gene calculated by the Cuffdiff program, and the cut-off value is set to *p*-value = 0.05. The number of SPRC-upregulated genes colored in red is 162, whose *p*-values are smaller than 0.05. The number of SPRC-downregulated genes colored in green is 108, whose *p*-values are smaller than 0.05. **(B)** The heatmap of the enrichment of the differentially expressed genes under the SPRC treatment comparing with the control condition whose log_2_(fold changes) are larger than +0.585 or smaller than –0.585. The enrichment illustrates a good triplicates situation and more SPRC-upregulated genes than SPRC-downregulated genes. The legend of the color bar displays the log_2_ values of the fold change of each replicate of each gene, from –2 to 2.

After the confirmation of the phenotypic alteration in the yeast upon the generation of H_2_S from SPRC, the transcriptomic analysis was performed to delineate the molecular mechanism underlying the yeast growth rate enhancement with the intracellular generation of H_2_S. The cell culture of the S288C strain supplemented with or without 2 mM SPRC for 16 h before the transcriptome assay was used. As depicted in Figure 2A, a total of 270 genes with significant differences in the expression levels with log_2_(fold change) values larger than 0.585 and less than −0.585 (*p* < 0.05) were obtained, among which 162 genes were upregulated, while the remaining 108 genes were downregulated, implying that the generated H_2_S from SPRC exerted the yeast growth enhancement effect mainly through the upregulated genes. This finding was further confirmed by the heatmap analysis (Figure 2B) and by the well-clustered triplicate samples of each group, in which the number of upregulated genes was larger than that of the downregulated genes upon the generation of H_2_S.

**FIGURE 3:**
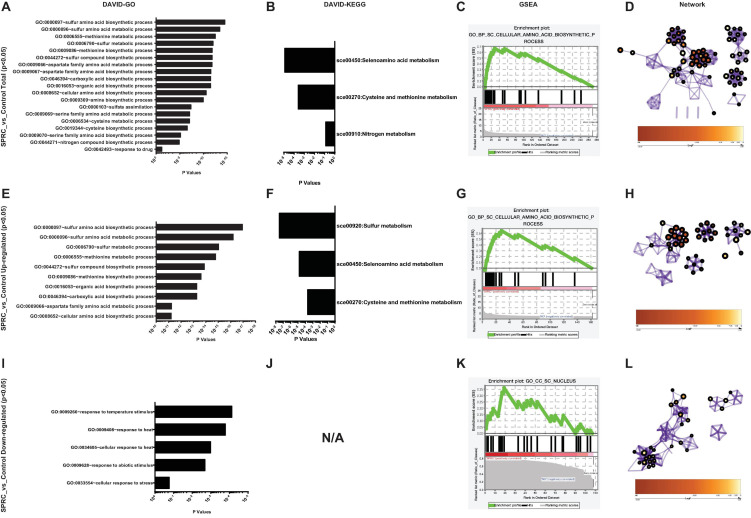
*S*-propargyl-cysteine (SPRC) elevating the expression of the genes involved in sulfur-amino-acid biosynthesis. **(A)** DAVID-GO analysis result of the total SPRC-significantly-influenced genes highlights the “GO:0000097” term of “sulfur amino acid biosynthetic process.” The horizontal axis shows the *p*-values of each enriched GO terms. **(B)** DAVID-KEGG analysis result of the total SPRC-significantly-influenced genes highlights the “sce00450” pathway of “Selenoamino acid metabolism.” The horizontal axis shows the *p*-values of each KEGG pathway. **(C)** The GSEA result showed the total SPRC-significantly-influenced genes are enriched into the Top-1-ranked GO term of “cellular amino acid biosynthetic process” with the enrichment score of 0.669408 and the nominal *p*-value of 0.00101833. **(D)** In Metascape-generated GO network, the total SPRC-significantly-influenced genes are mainly enriched in the GO terms related to sulfur-amino-acid biosynthetic process with a relatively small logP-value (Table 1). **(E)** DAVID-GO analysis result of the SPRC-upregulated genes. **(F)** DAVID-KEGG analysis result of the SPRC-upregulated genes. **(G)** The GSEA result showed the SPRC-upregulated genes. **(H)** In Metascape-generated GO network, the SPRC-upregulated are mainly enriched in the GO terms related to sulfur-amino-acid biosynthetic process with a small logP-value (Table 2). **(I)** DAVID-GO analysis result of the SPRC-downregulated genes. **(J)** DAVID-KEGG analysis result of the SPRC-downregulated genes fail to be enriched in any KEGG pathway even when the “classification stringency” is set to “lowest,” which implies that the SPRC-downregulated genes are not pathway-directed. **(K)** The GSEA result showed the SPRC-downregulated genes. **(L)** In Metascape-generated GO network, the SPRC-downregulated are mainly enriched in the GO terms related to energy and oxidation processes with a relatively large logP-values (Table 3).

Using these 270 genes with significant differences in their expression levels, including both the upregulated and downregulated genes (Supplementary Table 5), the functional information analysis was performed using DAVID-Gene Ontology. As illustrated in Figure 3A, these genes were significantly enriched into the sulfur-amino-acid-related GO terms, including the “sulfur amino acid biosynthetic process” and the “sulfur amino acid metabolic process.” A similar conclusion was reached in the DAVID-KEGG pathway analysis, in which the “seleno-amino acid metabolism” and the “cysteine and methionine metabolism” were significantly enriched (Figure 3B). Besides, these 270 genes together with their expression values were processed using GSEA, which revealed “cellular amino acid biosynthetic process” as the top-enriched GO term with a high enrichment score of over 0.6, thereby corroborating the results of the DAVID analysis (Figure 3C). The above results were supported by an additional analysis conducted using Metascape, in which the top 10 nodes in the darkest red with the highest *p*-values, including the node number 1–9 and node 97, were clustered within the networks, including the leading GO terms of “sulfur-amino-acids metabolic process” and “sulfur-compound biosynthetic process” with low *p*-values (Figure 3D and Table 1). Furthermore, all 10 enriched terms were related to the amino acid biological process (Figure 3D and Table 1).

**TABLE 1:** The information of the nudes clustered in Figure 3D.

Nude ID	#Gene hit list	Description	GO term	Group ID	LogP	STDV	*Z* score
97	21	Sulfur amino acid metabolic process	GO:0000096	1	−15.4057	1.774673	12.98227
1	31	Sulfur compound metabolic process	GO:0006790	1	−13.9626	2.10796	10.57599
2	35	Alpha-amino acid metabolic process	GO:1901605	1	−13.8483	2.218996	10.18649
3	18	Methionine metabolic process	GO:0006555	1	−13.8105	1.653897	12.45484
4	17	Sulfur amino acid biosynthetic process	GO:0000097	1	−12.7771	1.610805	11.86046
5	19	Aspartate family amino acid biosynthetic process	GO:0009067	1	−12.5129	1.695504	11.19716
6	21	Aspartate family amino acid metabolic process	GO:0009066	1	−12.4005	1.774673	10.7511
7	16	Methionine biosynthetic process	GO:0009086	1	−12.1777	1.566104	11.6122
8	27	Alpha-amino acid biosynthetic process	GO:1901607	1	−12.1738	1.985399	9.84303
9	22	Sulfur compound biosynthetic process	GO:0044272	1	−12.1097	1.812413	10.38678

In particular, the 162 upregulated genes among the total 270 genes were analyzed to specifically reveal the properties of the transcriptomic upregulation of these genes. The “sulfur amino acid biosynthetic process” remained the top-enriched GO term in this analysis as well (Figure 3E), similar to the one in the analysis of the total number of genes (Figure 3A). However, the *p*-values of the enriched GO terms of the upregulated genes were much smaller than the ones of the total 270 genes, which implied higher credibility of the result for the GO terms of the 162 upregulated genes. A similar phenomenon was demonstrated in the KEGG analysis of the enriched pathways of upregulated genes (Figure 3F), with the obtained smaller *p*-values being more convincing than the larger ones obtained in the analysis of total genes (Figure 3B). The subsequent GSEA analysis of the upregulated genes (Figure 3G) also highlighted the “cellular amino acid biosynthetic process” as the top-enriched GO term, thereby corroborating the GSEA result obtained in the analysis of the total 270 genes (Figure 3C). These 162 genes were then processed in the Metascape network analysis, in which they were enriched to the “sulfur amino acid metabolic process” pathway (Figure 3H and Table 2) with a much smaller *p*-value compared to the one generated from the analysis of total genes (Figure 3D and Table 1), implying higher credibility of the enrichment result for the SPRC-upregulated genes compared to that for the total genes.

**TABLE 2:** The information of the nudes clustered in Figure 3H.

Nude ID	#Gene hit list	Description	GO term	Group ID	LogP	STDV	*Z* score
2	20	Sulfur amino acid metabolic process	GO:0000096	1	−18.4759	2.758578	16.33831
63	17	Methionine metabolic process	GO:0006555	1	−16.1884	2.57224	15.49488
62	26	Sulfur compound metabolic process	GO:0006790	1	−15.0493	3.072389	11.97794
61	16	Sulfur amino acid biosynthetic process	GO:0000097	1	−14.9489	2.504733	14.7213
60	20	Sulfur compound biosynthetic process	GO:0044272	1	−14.2937	2.758578	12.68996
59	15	Methionine biosynthetic process	GO:0009086	1	−14.1238	2.434163	14.34356
58	28	Alpha-amino acid metabolic process	GO:1901605	1	−14.0886	3.162759	11.02434
57	22	Alpha-amino acid biosynthetic process	GO:1901607	1	−12.459	2.87105	10.77938
56	30	Cellular amino acid metabolic process	GO:0006520	1	−12.1864	3.247042	9.588036
55	17	Aspartate family amino acid metabolic process	GO:0009066	1	−11.9671	2.57224	11.48193

**TABLE 3:** The information of the nudes clustered in Figure 3L.

Nude ID	#Gene hit list	Description	GO term	Group ID	LogP	STDV	*Z* score
5	13	Energy derivation by oxidation of organic compounds	GO:0015980	1	−5.63141	3.492238	6.400214
69	21	Oxidation-reduction process	GO:0055114	1	−4.81791	4.219232	5.068667
68	13	Generation of precursor metabolites and energy	GO:0006091	1	−4.30953	3.492238	5.115331
67	6	Cellular respiration	GO:0045333	1	−2.01229	2.470529	3.135611

In contrast to the above results, the analyzed 108 downregulated genes presented opposite ones. These genes were enriched into certain GO terms such as “response to temperature stimulus” (Figure 3I) with considerably high *p*-values compared to those obtained from the total genes, particularly those obtained from upregulated genes. Furthermore, the KEGG terms of the downregulated genes simply failed to be enriched onto any pathway even when the “classification stringency” settings in the DAVID system were set to “lowest,” which implied that the downregulated genes had less significant biological importance compared to the upregulated ones upon the generation of H_2_S (Figure 3J). This finding was supported by the GSEA analysis with “CC_SC_nucleus” as the top-enriched GO term with a low enrichment score and a low ranked list metric (Figure 3K). The network analysis also illustrated the enriched nodes of temperature-stimulus-related in the light color region of higher *p*-values (Figure 3L). In general, the H_2_S generation from SPRC catabolism in yeast occurred with the upregulation of the expressions of genes in sulfur amino acid-related pathways, which was accompanied by the phenotype of enhanced growth rate in the yeast.

### H_2_S Presumed to Be Generated Because of the Catabolic Action of Cystathionine-γ-Lyase, a Potential Enzyme for SPRC Metabolism

**FIGURE 4:**
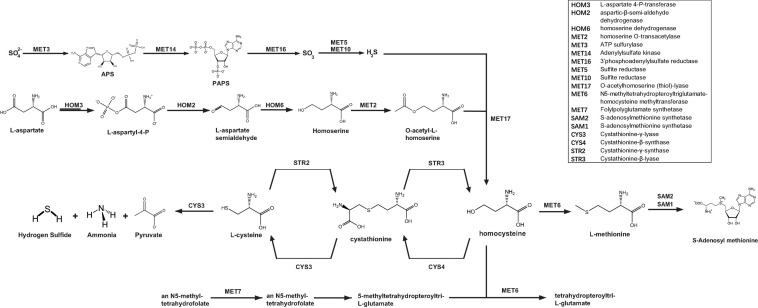
“Sulfur amino acid biosynthesis superpathway” of *S. cerevisiae*. The re-organized sulfur amino acid biosynthesis superpathway of *S. cerevisiae* illustrates the sulfur metabolism in the yeast cells. The inorganic sulfur is initially metabolized in the sub-branch pathway by *MET3* and the subsequent enzyme genes (*MET14*, *MET16*, *MET5*, and *MET10*) and then generate H_2_S, which will be synthesized to be homocysteine synthesized from the non-sulfur L-aspartate in the sub-branch pathway by *HOM3* and the downstream enzyme genes (*HOM2*, *HOM6*, *MET2*, and *MET17*). For the sub-branch pathway begun with *MET7*, the structure of the initial substrate, an *N*^5^-methyl-tetrahydrofolate, is far from the one of SPRC, and this sub-branch generate no H_2_S. Whereas the substrates in the sub-branch pathway (*CYS3*, *CYS4*, *STR2*, and *STR3*) are all sulfur-amino-acids and are structurally similar to SPRC, and also whereas *CYS3* is capable to catalyze L-cysteine to generate H_2_S, this sub-branch is expected to be the metabolism pathway to SPRC and CYS3 is selected to be the catabolic enzyme candidate to SPRC for the consequent assays.

Although the analyses conducted in the present study revealed that the sulfur amino acid-related pathways were upregulated upon H_2_S generation from SPRC catabolism, a metabolic map based on the *Saccharomyces* Genome Database (SGD) is summarized in Figure 4 for comprehension, as the sulfur amino acid biosynthesis in *S. cerevisiae* involves numerous enzymes required for *de novo* biosynthesis of sulfur amino acids as well as for transferring the organic sulfur among the metabolites. As illustrated in the upper portion of Figure 4, only a reduced sulfur atom can be incorporated into carbon chains via sulfate adenylation to lower the electron potential and a further reduction by the oxidation of NADPH. Since this process of assimilation aimed at reducing the electro-redox state of the inorganic sulfate, the enzymes involved in the activation of this process were excluded from being the SPRC-consuming protein candidates. Subsequently, S^2–^ could be incorporated into four-carbon chain homocysteine, which is interconverted by the *trans*-sulfuration pathway into L-cysteine via the intermediary formation of cystathionine. As depicted in Figure 4, SPRC is an analog of L-cysteine, suggesting that cystathionine-γ-lyase could be responsible for catabolizing SPRC and generating the H_2_S detected in the yeast. Cystathionine-γ-lyase is one of the three enzymes (cystathionine β-synthase and 3-MST being the other two enzymes) involved in producing H_2_S (Yu et al., 2014). In addition to catalyzing L-cystathionine into L-cysteine (EC 4.4.1.1, ID: RXN-15130), cystathionine-γ-lyase causes the further breakdown of L-cysteine into pyruvate, NH_3_, and H_2_S (EC 4.4.1.1/4.4.1.28 ID: LCYSDESULF-RXN). Therefore, cystathionine-γ-lyase was preliminarily considered for application as the catabolic enzyme, as it was presumed to be involved in the generation of H_2_S by catabolizing SPRC and thereby enhancing the yeast growth rate.

### H_2_S Confirmed to Be Generated Through the Catabolic Action of Yeast Cystathionine-γ-Lyase

**FIGURE 5:**
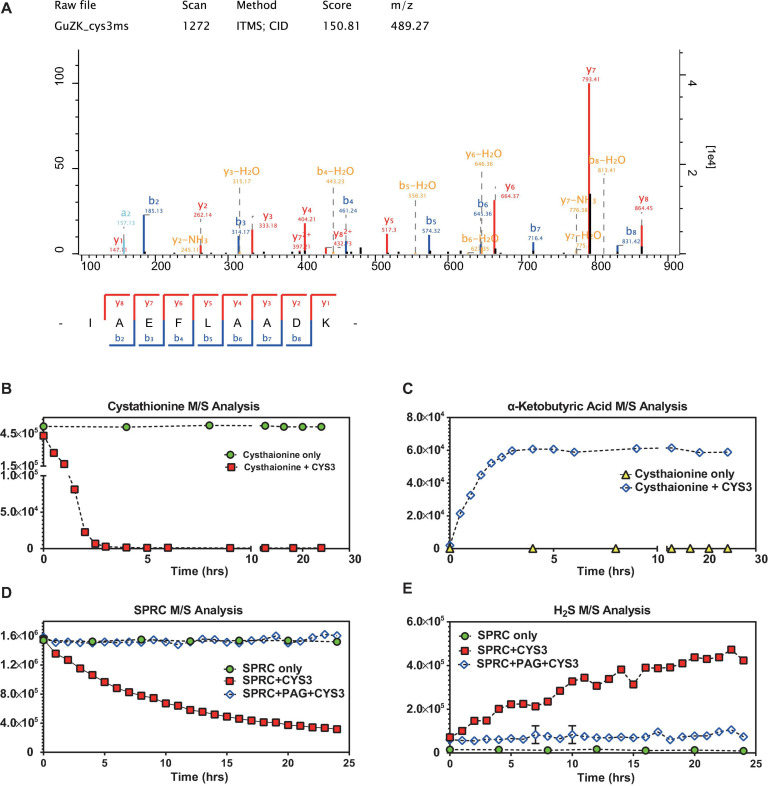
*S*-propargyl-cysteine catalyzed by yeast cystathionine-γ-lyase. **(A)** The mass spectrometry analysis confirmed the identity of Cys3p (cystathionine-γ-lyase in S288C) purified from *E. coli* lysis. The sequence of the peptide of Cys3p has been identified as “IAEFLAADK,” which matched to the sequence of Cys3p by NCBI-BLASTp. The horizontal axis shows the molecular weight, and the vertical axis shows the signal intensity. **(B)** The mass spectrometry analysis showed relatively rapid decreasing signals of cystathionine when cystathionine was being catalyzed by purified Cys3p from 0 to 24 h, and non-decreasing signals of cystathionine without the Cys3p incubation. **(C)** The mass spectrometry analysis showed relatively rapid increasing signals of α-ketobutyric acid when cystathionine was being catalyzed by purified Cys3p from 0 to 24 h, and non-increasing signals of α-ketobutyric acid without the Cys3p incubation. **(D)** The mass spectrometry analysis showed relatively stable decreasing signals of SPRC when SPRC was being catalyzed by purified Cys3p from 0 to 24 h, and non-decreasing signals of SPRC when the catalysis inhibited by PAG. **(E)** The mass spectrometry analysis showed relatively stable increasing signals of H_2_S when SPRC was being catalyzed by purified Cys3p from 0 to 24 h, and non-increasing signals of H_2_S when the catalysis inhibited by PAG. **(B–E)** The horizontal axes indicate the incubation time (0–24 h). The vertical axes indicate the M/S signals of the detected molecules, including cystathionine, α-ketobutyric acid, SPRC and H_2_S. The data of triplicates was represented as mean ± SD.

The cystathionine-γ-lyase of *S. cerevisiae* (abbreviated as *CYS3*) was expressed in *E. coli* (Supplementary Figures 2–5), purified, and then subjected to mass spectrometry identification (depicted in Figure 5A and Supplementary Table 4). Although Cys3p catalyzed both cystathionine and L-cysteine as substrates, the canonical one, i.e., cystathionine, was selected to be used as the control molecule for evaluating the enzymatic activity (Yamagata et al., 1993). In order to determine whether the purified Cys3p retained its enzymatic function, the catabolism of cystathionine was monitored in the presence or absence of Cys3p. As illustrated in Figures 5B,C, in the absence of Cys3p, cystathionine was chemically-stable, while α-ketobutyric acid, a well-recognized product of Cys3p catalysis on cystathionine (Yamagata et al., 1993), was undetectable. However, when Cys3p was added, the M/S signals of cystathionine decreased rapidly, while the concentration of α-ketobutyric acid increased, demonstrating the functional catalytic enzyme activity of purified Cys3p. Subsequently, SPRC degradation and H_2_S generation could be observed upon Cys3p serving (Figures 5D,E), in contrast to the absence of it. Moreover, the Cys3p inhibitor PAG could specifically inhibit such enzymatic activity (Wang et al., 2009), which again confirmed the enzymatic activity of purified Cys3p. Taken together, these results confirmed the additional generation of H_2_S upon the service of Cys3p *in vitro*, suggesting that Cys3p was an H_2_S-generating enzyme in yeast, which acted by catalyzing the substrate SPRC, and implying that the growth rate of yeast could be reduced by inhibiting the activity of this enzyme.

### Cystathionine-γ-Lyase Identified as a Potential Enzyme for Inhibition Owing to Its High Conservation Throughout Evolution

**FIGURE 6:**
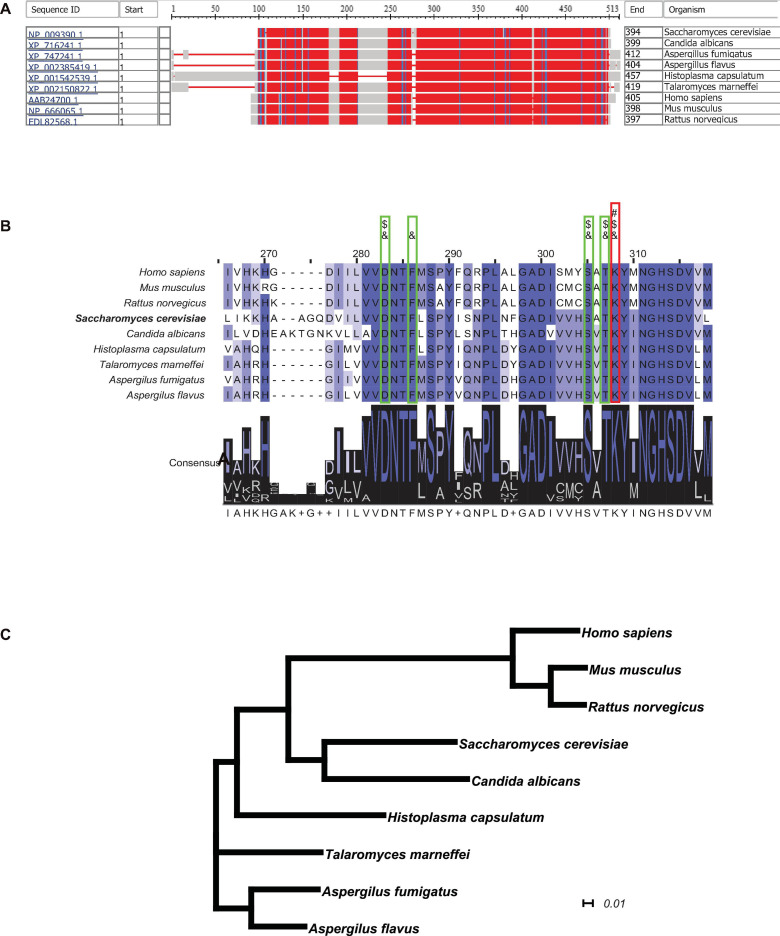
Cystathionine-γ-lyase is a highly conserved enzyme throughout evolution. **(A)** The NCBI-COBALT (Constraint-based Multiple Alignment Tool) alignment display cystathionine-γ-lyase homologs from multiple species. The red parts show the highly conserved domains and the blue parts for less conserved domains, gray parts for no consensus of conservation. **(B)** The Conserved Protein Domain Family alignment revealed the key catalytic residues. The PID (Percentage Identity Color) codes for amino acid residue are as following: darkest cyan: the residues according to the percentage of the residues in each column that agree with the consensus sequence of a level of 80% or more; middle cyan between 60 and 80%; lightest cyan between 40 and 60%; no color when the consensus level below 40%. A “#” symbol represents a Schiff-base forming lysine residue at which PLP forms an internal aldimine bond. An “&” symbol represents an adjacent residue forming a hydrogen bond with the phosphate group of PLP for anchoring. A “$” symbol represents an adjacent residue involved in substrate-cofactor accommodation. **(C)** The phylogenetic analysis showed the evolutionary conservatism of the cystathionine-γ-lyase among the 12 species. The plotting scale shows the evolutionary distance of 0.1 *K*_nuc_.

In order to obtain further insight into the possibility of using cystathionine-γ-lyase as a metabolic enzyme candidate for suppressing the generation of H_2_S and inhibiting fungal growth rate, the NCBI-COBALT (Constraint-based Multiple Alignment Tool) analysis was performed to examine the conservation of cystathionine-γ-lyase across different species. The full-length protein sequences of cystathionine-γ-lyase from 9 species, including 6 of pathogenic fungi and 3 of mammals, were retrieved and displayed in a bird’s-eye scope. In Figure 6A, the parts with high degrees of sequence homology are depicted in red, while the parts with a relatively low level of homology are depicted in blue or gray. As hypothesized, cystathionine-γ-lyase was a highly conserved metabolic protein, as evidenced by all species sharing large parts of the protein sequence of this enzyme as the conserved domains. The sequences of the enzyme in the nine species were further clustered using the ClustalΩ algorithm, generating the consensus across the species, which are depicted in Figure 6B. The Schiff-base-forming lysine residue of *S. cerevisiae* labeled “#” (Messerschmidt et al., 2003) is the site at which PLP formed an internal aldimine bond that was invariably conserved across all nine species. In addition, the adjacent residues forming hydrogen bonds with the phosphate group of PLP for anchoring were labeled “&” (Messerschmidt et al., 2003), and the ones involved in the substrate–cofactor accommodation were labeled “$” (Clausen et al., 1996). These residues were also completely identical among all nine species, demonstrating the motifs conserved through evolution. Moreover, for visualizing the evolutionary association among the selected species, a phylogenetic tree was generated using CLUSTALW (Figure 6C), which illustrated a narrow range of evolutionary distances of cystathionine-γ-lyase, particularly among the fungal species. In summary, cystathionine-γ-lyase was observed to be highly conserved across species, which suggested the importance of this enzyme in biological activities, particularly in those involved in the regulation of the growth of pathogenic fungi via influencing the sulfur metabolism for the intracellular generation of H_2_S. In addition to producing the phenotype of yeast growth enhancement, the conservation of cystathionine-γ-lyase is expected to be useful for developing anti-fungal drugs in the future by serving as an enzyme candidate for inhibition.

## Discussion

Fungal infections lead to millions of cases of infection and deaths every year. The growth of fungi is subject to several factors, one of which is gaseous signaling molecules. H_2_S is a well-recognized and extensively studied gaseous signaling molecule that ranks third after the signaling molecules NO and CO. H_2_S is reported to be involved in physiological functions related to cardiovascular protection, such as homeostasis and smooth muscle relaxation. Moreover, since H_2_S generation is a common phenomenon during the fermentation of yeast, it was reasonable to hypothesize that H_2_S possibly regulates the growth of yeast and/or the other species of fungi. However, studies on the potential effect of H_2_S on fungi encounters two limitations. One is that the H_2_S generated from sulfide salts, such as NaHS, is gaseous and unstable for using dosage control, and owing to the exogenous generation, this H_2_S exhibits properties different from the ones described for the endogenously-generated H_2_S during yeast fermentation. The other limitation is that little is known regarding the effect of the phenotypic or transcriptomic alternations on the fungal growth enhancement by H_2_S. Therefore, in the present study, SPRC, which is a stable donor that gently produces H_2_S intracellularly under catalytic action *in vivo*, was employed to unravel the mechanism operating in the budding yeast *S. cerevisiae* S288C that was used as a fungal model.

The results demonstrated that the growth rate of yeast was enhanced upon the intracellular generation of H_2_S, while the exogenously served H_2_S offered little contribution. In addition, the transcriptomic study preliminarily revealed that the intracellularly-generated H_2_S was produced with the elevation of the expression levels of the genes associated with the metabolism of cysteine-like substrates in the sulfur-amino-biosynthesis metabolism pathway. Finally, Cys3p was revealed and confirmed to be capable of catalyzing SPRC for H_2_S production *in vitro*, which led to the inference that *CYS3* could serve to influences the growth rate of yeast and even that of the other fungi by regulating the sulfur metabolism in these fungi. These findings contribute a possibility to the pharmacological industry that *CYS3* has become worth of the development of anti-fungal drugs against fungal infections and providing relief to patients with fungal infections and diseases.

## Data Availability Statement

The datasets generated for this study can be found in online repositories. The names of the repository/repositories and accession number(s) can be found below: https://www.ncbi.nlm.nih.gov/, PRJNA714796.

## Author Contributions

ZG designed the total topic and all the experiments and took in charge of almost all the experiments and the data analyses. YS took in charge of the purification of Cys3p of budding yeast. FW took in charge of the direction on the data analyses. XW took in charge of the manuscript preparation. All the authors contributed to the article and approved the submitted version.

## Conflict of Interest

The authors declare that the research was conducted in the absence of any commercial or financial relationships that could be construed as a potential conflict of interest.

## Footnotes

1
https://www.bioinformatics.babraham.ac.uk/projects/fastqc/

2
https://www.bioinformatics.babraham.ac.uk/projects/trim_galore/

3
https://support.illumina.com/sequencing/sequencing_software/igenome.html
