# Corrigendum: Mechanism of Growth Regulation of Yeast Involving Hydrogen Sulfide From *S*-Propargyl-Cysteine Catalyzed by Cystathionine-γ-Lyase

**DOI:** 10.3389/fmicb.2021.761240

**Published:** 2021-09-24

**Authors:** Zhongkai Gu, Yufan Sun, Feizhen Wu, Xiaomo Wu

**Affiliations:** ^1^The Institute of Biomedical Sciences, Fudan University, Shanghai, China; ^2^Key Laboratory of Medical Molecular Virology of Ministries of Education and Health, Department of Medical Microbiology, School of Basic Medical Sciences, Fudan University, Shanghai, China; ^3^Dermatology Institute of Fuzhou, Dermatology Hospital of Fuzhou, Fuzhou, China

**Keywords:** hydrogen sulfide, H_2_S metabolism, fungal growth rate, SPRC, cystathionine-γ-lyase, fungal growth

Xiaomo Wu was not included as an author in the published article. Her affiliation is “Dermatology Institute of Fuzhou, Dermatology Hospital of Fuzhou, Fuzhou, China”. She is a corresponding author of the article. The corrected Author Contributions Statement appears below.

“ZG designed the total topic and all the experiments and took in charge of almost all the experiments and the data analyses. YS took in charge of the purification of Cys3p of budding yeast. FW took in charge of the direction on the data analyses. XW took in charge of the manuscript preparation. All the authors contributed to the article and approved the submitted version.”

In the original article, we neglected to include the funder Key Clinical Specialty Discipline Construction Program of Fuzhou, 201807111, and Clinical Medicine Center Construction Program of Fuzhou, 2018080309 to Xiaomo Wu. The corrected Funding statement appears below.

“The study was funded in part by a grant from the National Key Basic Research Project of China [grant number: 2013CB531603]. This work was supported by Key Clinical Specialty Discipline Construction Program of Fuzhou (201807111) and Clinical Medicine Center Construction Program of Fuzhou (2018080309) to XW.”

The authors apologize for this error and state that this does not change the scientific conclusions of the article in any way. The original article has been updated.

## Publisher's Note

All claims expressed in this article are solely those of the authors and do not necessarily represent those of their affiliated organizations, or those of the publisher, the editors and the reviewers. Any product that may be evaluated in this article, or claim that may be made by its manufacturer, is not guaranteed or endorsed by the publisher.

