# Supplementary material for: Mechanism of Growth Regulation of Yeast Involving Hydrogen Sulfide From S-Propargyl-Cysteine Catalyzed by Cystathionine-γ-Lyase
Source: Front Microbiol. 2021 Jul 2;12:679563. doi: 10.3389/fmicb.2021.679563 (PMC8285084; doi:10.3389/fmicb.2021.679563)
Supplement: Supplementary file 1 [file Data_Sheet_1.DOCX]

Supplementary

Figure 1. The standard curve of *S. cerevisiae* S288C cells in SD medium for the cell density conversion. The OD_595nm_ data are referred to Supplemental Table 1 below. The formula shows the conversion between cell densities and OD_595nm_ values.


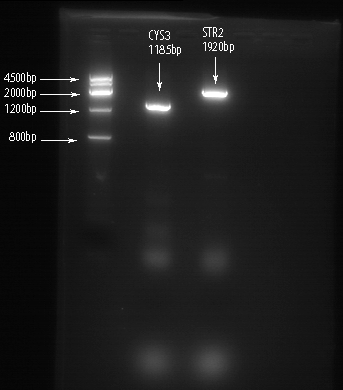


Figure 2. The photo of 1% agarose gel showed the CYS3 fragments which were just amplified from the S288C gDNA by PCR. The band of CYS3 clearly displayed on the gel at the position where was 1185 bp, just near the 1200 bp band of the ladder. The PCR product was ready for the consequent sub-cloning assay.


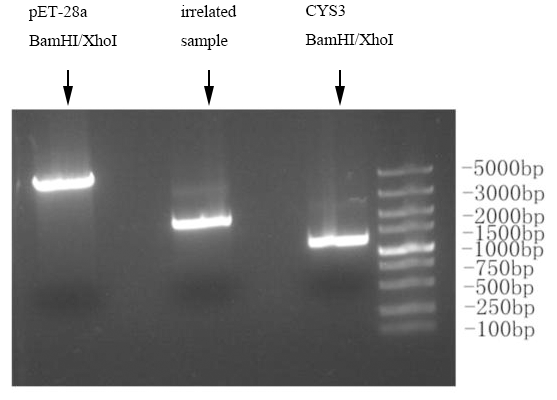


Figure 3. The photo to the 1% agarose gel clearly displayed the enzyme digestion results. The band of the linearized pET-28a vector plasmid was obviously visible, which had been digested by BamHI and XhoI. In addition, the band of the digested CYS3 fragments was also located at the right position. Therefore, the linearized vector plasmid and the CYS3 fragments were ready for the subsequent ligation assay in order to subclone the gene into the plasmid.


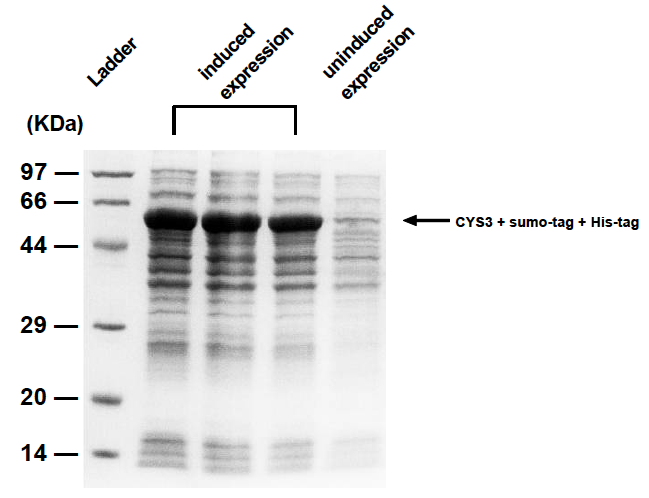


Figure 4. The SDS-PAGE above illustrated the large difference of the Cys3p protein expression between under an IPTG-inducement condition and under a non-inducement one. The wide bands clearly demonstrated the induced expression of Cys3p together with the mass weights of the His6-tag and a SUMO-tag, both of which would be cleaved from Cys3p during the following purification procedures.


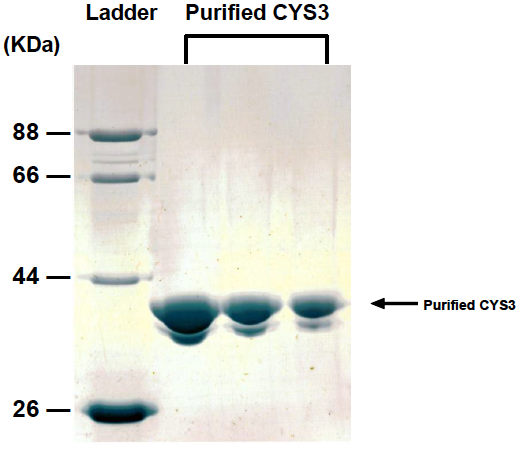


Figure 5. In the SDS-PAGE picture above, the bands of the purified Cys3p were clearly illustrated reflected by a background with little noise, which revealed a fact of the successful purification of Cys3p. The 3 strong bands of different loading doses were just at the right place where Cys3p should be according to its molecular weight. The 3 weak bands below the 3 strong ones indicated the partially degraded Cys3p, which would be confirmed by the following mass spectrometry detection.

| Time (hours) | Control | | | 0.1mM SPRC | | | 0.5mM SPRC | | | 1mM SPRC | | | 2mM SPRC | | | 3mM SPRC | | | 4mM SPRC | | | 5mM SPRC | | | 7.5mM SPRC | | | 10mM SPRC | | |
| --- | --- | --- | --- | --- | --- | --- | --- | --- | --- | --- | --- | --- | --- | --- | --- | --- | --- | --- | --- | --- | --- | --- | --- | --- | --- | --- | --- | --- | --- | --- |
| 0 | 0.0764 | 0.0771 | 0.0772 | 0.0764 | 0.0775 | 0.085 | 0.0766 | 0.0777 | 0.0761 | 0.0776 | 0.0769 | 0.0771 | 0.0773 | 0.0788 | 0.0772 | 0.0764 | 0.078 | 0.0775 | 0.0766 | 0.0777 | 0.0771 | 0.0764 | 0.0763 | 0.0773 | 0.076 | 0.0766 | 0.0762 | 0.0758 | 0.0823 | 0.0764 |
| 10 | 0.0817 | 0.0825 | 0.0813 | 0.0808 | 0.083 | 0.0895 | 0.0812 | 0.0812 | 0.0829 | 0.0831 | 0.0814 | 0.0829 | 0.0835 | 0.085 | 0.0865 | 0.0837 | 0.0856 | 0.0832 | 0.0833 | 0.0853 | 0.0831 | 0.0836 | 0.0842 | 0.0833 | 0.0845 | 0.0823 | 0.082 | 0.0819 | 0.0817 | 0.0808 |
| 12 | 0.0895 | 0.0911 | 0.0906 | 0.0884 | 0.0906 | 0.099 | 0.0906 | 0.0915 | 0.0942 | 0.0936 | 0.0908 | 0.095 | 0.0982 | 0.0992 | 0.0996 | 0.0975 | 0.1009 | 0.0973 | 0.097 | 0.0979 | 0.0976 | 0.0975 | 0.0984 | 0.0974 | 0.0959 | 0.0939 | 0.0938 | 0.0914 | 0.0903 | 0.0894 |
| 14 | 0.1205 | 0.1175 | 0.1053 | 0.1208 | 0.1185 | 0.1219 | 0.1285 | 0.1259 | 0.1227 | 0.1412 | 0.1276 | 0.1247 | 0.1459 | 0.144 | 0.1347 | 0.1485 | 0.1434 | 0.1347 | 0.1441 | 0.1414 | 0.1354 | 0.1413 | 0.1376 | 0.1305 | 0.1301 | 0.1222 | 0.1211 | 0.1166 | 0.1188 | 0.116 |
| 16 | 0.1908 | 0.1915 | 0.1798 | 0.1913 | 0.193 | 0.1976 | 0.205 | 0.2022 | 0.1967 | 0.2203 | 0.2087 | 0.2034 | 0.2372 | 0.2343 | 0.2192 | 0.2375 | 0.2282 | 0.2178 | 0.2286 | 0.223 | 0.2165 | 0.2206 | 0.2166 | 0.2068 | 0.2031 | 0.1887 | 0.1854 | 0.1732 | 0.1746 | 0.1729 |
| 18 | 0.2984 | 0.2984 | 0.2829 | 0.2983 | 0.3015 | 0.2976 | 0.3166 | 0.3126 | 0.3 | 0.3292 | 0.3165 | 0.3087 | 0.3416 | 0.3395 | 0.3232 | 0.3361 | 0.3316 | 0.3197 | 0.3326 | 0.3185 | 0.3144 | 0.3101 | 0.3063 | 0.2946 | 0.2834 | 0.2665 | 0.2644 | 0.2481 | 0.2546 | 0.2508 |
| 20 | 0.4339 | 0.4362 | 0.424 | 0.4303 | 0.4385 | 0.4402 | 0.4497 | 0.4472 | 0.4448 | 0.4617 | 0.448 | 0.449 | 0.4605 | 0.4646 | 0.4506 | 0.4442 | 0.4425 | 0.4362 | 0.4332 | 0.4265 | 0.4257 | 0.4079 | 0.4128 | 0.4097 | 0.3707 | 0.3656 | 0.3738 | 0.3286 | 0.3247 | 0.3219 |
| 22 | 0.5953 | 0.5994 | 0.5876 | 0.5956 | 0.6004 | 0.605 | 0.6119 | 0.6076 | 0.6063 | 0.616 | 0.5992 | 0.598 | 0.6083 | 0.6104 | 0.5882 | 0.5766 | 0.5797 | 0.5613 | 0.5535 | 0.5515 | 0.5388 | 0.5259 | 0.5306 | 0.5229 | 0.4716 | 0.4664 | 0.4734 | 0.415 | 0.4158 | 0.4091 |
| 24 | 0.7108 | 0.715 | 0.7062 | 0.7074 | 0.747 | 0.7268 | 0.7288 | 0.7395 | 0.7181 | 0.7187 | 0.7041 | 0.7035 | 0.7083 | 0.7085 | 0.6896 | 0.6768 | 0.6763 | 0.6616 | 0.6525 | 0.6495 | 0.6402 | 0.63 | 0.6369 | 0.6243 | 0.5786 | 0.5729 | 0.5676 | 0.5235 | 0.5206 | 0.5126 |

Table 1. The original OD_595nm_ values of yeast cells when growing in the medium containing SPRC of different concentrations from 0 to 24 h

| Time (hours) | Control | | | 1uM NaHS | | | 2uM NaHS | | | 4uM NaHS | | | 6uM NaHS | | | | 8uM NaHS | | | |
| --- | --- | --- | --- | --- | --- | --- | --- | --- | --- | --- | --- | --- | --- | --- | --- | --- | --- | --- | --- | --- |
| 0 | 0.0764 | 0.0771 | 0.0772 | 0.0764 | 0.0775 | 0.085 | 0.0766 | 0.0777 | 0.0761 | 0.0776 | 0.0769 | 0.0771 | 0.0773 | 0.0788 | 0.0772 | 0.0764 | | 0.078 | 0.0775 |  |
| 10 | 0.0817 | 0.0825 | 0.0813 | 0.0808 | 0.083 | 0.0895 | 0.0812 | 0.0812 | 0.0829 | 0.0831 | 0.0814 | 0.0829 | 0.0835 | 0.085 | 0.0865 | 0.0837 | | 0.0856 | 0.0832 |  |
| 12 | 0.0895 | 0.0911 | 0.0906 | 0.0884 | 0.0906 | 0.099 | 0.0906 | 0.0915 | 0.0942 | 0.0936 | 0.0908 | 0.095 | 0.0982 | 0.0992 | 0.0996 | 0.0975 | | 0.1009 | 0.0973 |  |
| 14 | 0.1205 | 0.1175 | 0.1053 | 0.1208 | 0.1185 | 0.1219 | 0.1285 | 0.1259 | 0.1227 | 0.1412 | 0.1276 | 0.1247 | 0.1459 | 0.144 | 0.1347 | 0.1485 | | 0.1434 | 0.1347 |  |
| 16 | 0.1908 | 0.1915 | 0.1798 | 0.1913 | 0.193 | 0.1976 | 0.205 | 0.2022 | 0.1967 | 0.2203 | 0.2087 | 0.2034 | 0.2372 | 0.2343 | 0.2192 | 0.2375 | | 0.2282 | 0.2178 |  |
| 18 | 0.2984 | 0.2984 | 0.2829 | 0.2983 | 0.3015 | 0.2976 | 0.3166 | 0.3126 | 0.3 | 0.3292 | 0.3165 | 0.3087 | 0.3416 | 0.3395 | 0.3232 | 0.3361 | | 0.3316 | 0.3197 |  |
| 20 | 0.4339 | 0.4362 | 0.424 | 0.4303 | 0.4385 | 0.4402 | 0.4497 | 0.4472 | 0.4448 | 0.4617 | 0.448 | 0.449 | 0.4605 | 0.4646 | 0.4506 | 0.4442 | | 0.4425 | 0.4362 |  |
| 22 | 0.5953 | 0.5994 | 0.5876 | 0.5956 | 0.6004 | 0.605 | 0.6119 | 0.6076 | 0.6063 | 0.616 | 0.5992 | 0.598 | 0.6083 | 0.6104 | 0.5882 | 0.5766 | | 0.5797 | 0.5613 |  |
| 24 | 0.7108 | 0.715 | 0.7062 | 0.7074 | 0.747 | 0.7268 | 0.7288 | 0.7395 | 0.7181 | 0.7187 | 0.7041 | 0.7035 | 0.7083 | 0.7085 | 0.6896 | 0.6768 | | 0.6763 | 0.6616 |  |

Table 2. The original OD_595nm_ values of yeast cells when growing in the medium containing NaHS of different concentrations from 0 to 24 h

Table 3. The OD_595nm_ values of the yeast cell samples for the standard curve measurement.

| OD_595nm_ | Value 1 | Value 2 | Value 3 | Mean |
| --- | --- | --- | --- | --- |
| 1x | 0.732 | 0.7962 | 0.7832 | 0.7705 |
| 0.8x | 0.6044 | 0.5874 | 0.5893 | 0.5937 |
| 0.6x | 0.4506 | 0.4689 | 0.4746 | 0.4647 |
| 0.4x | 0.3244 | 0.3347 | 0.3358 | 0.3316 |
| 0.2x | 0.2098 | 0.2149 | 0.2097 | 0.2115 |
| 0x (Blank) | 0.0789 | 0.0766 | 0.0778 | 0.0778 |

Table 4. The mass spectrometry result showed the detected peptides in the Cys3p solution. In the MS signal table below, the 22 fragments of YAL012W (CYS3) was detected. The molecular mass of YAL012W had been identified as 42.542 kDa, which was just the one of Cys3p. The analyzed sequence length was 394, which matched the amino acid length of Cys3p. The most confident evidence came from the figures of intensities, among which the ones of YAL012W owned a number of 1.44×10^10^. The statistics above implied a fact that the protein of Cys3p had occupied a dominant position among all the detected protein members in the solution by its intensity figure which was much higher than the ones of others by two to three orders of magnitude. Moreover, the statistics also revealed another fact that the protein with the weak bands above were the degraded parts of Cys3p, or they should have been offered a very high intensity number. However, in the statistics, the second intensity to YAL012W was 2.28×10^8^ of YLR135W, whose molecular weight was 84.361 and much heavier than YAL012W but not visible on the SDS-PAGE gel above. Therefore, the 3 weak bands were believed to belong to the partially-degraded Cys3p, which additionally confirmed the purity of Cys3p in the total solution after the series of purifications.

| Protein IDs | Peptides | Sequence coverage [%] | Mol. weight [kDa] | Sequence length | Intensity |
| --- | --- | --- | --- | --- | --- |
| YAL012W | 22 | 68.8 | 42.542 | 394 | 1.44E+10 |
| YBR140C | 1 | 0.7 | 350.98 | 3092 | 1.32E+07 |
| YCL061C | 1 | 1.4 | 124.32 | 1096 | 2.16E+06 |
| YDR510W | 4 | 40.6 | 11.597 | 101 | 3.37E+07 |
| YGL145W | 1 | 1.7 | 81.165 | 701 | 5.24E+07 |
| YGR023W | 1 | 1.5 | 57.527 | 551 | 6.37E+06 |
| YIL144W | 1 | 1.4 | 80.486 | 691 | 2.70E+06 |
| YLR135W | 1 | 1.2 | 84.361 | 748 | 2.28E+08 |
| YNL047C | 1 | 1.5 | 74.784 | 656 | 1.84E+05 |
| YNL123W | 1 | 2.3 | 110.88 | 997 | 0.00E+00 |
| YNL138W | 1 | 3.4 | 57.521 | 526 | 4.39E+06 |
| YNL272C | 1 | 1.4 | 84.651 | 759 | 7.85E+07 |
| YOR008C | 1 | 3.7 | 39.27 | 378 | 3.15E+06 |

Table 5: The table of the significantly-affected genes under the treatment of SPRC at 2 mM at 16 h. All the p-values are below 0.05. Log2(fold change) ≥ 0.585 or ≤ -0.585.

| Index | Gene | Gene ID | Control FPKM_mean | SPRC FPKM_mean | log2(fold change) | p_value |
| --- | --- | --- | --- | --- | --- | --- |
| 1 | DAN1 | YJR150C | 5.87903 | 128.679 | 4.45206 | 5.00E-05 |
| 2 | HES1 | YOR237W | 4.08919 | 38.6125 | 3.23918 | 5.00E-05 |
| 3 | TIR1 | YER011W | 91.0365 | 481.977 | 2.40445 | 5.00E-05 |
| 4 | SEO1 | YAL067C | 2.93137 | 13.8041 | 2.23545 | 5.00E-05 |
| 5 | YOL163W | YOL163W | 2.81495 | 12.7183 | 2.17573 | 0.04255 |
| 6 | MET3 | YJR010W | 134.259 | 513.383 | 1.93502 | 5.00E-05 |
| 7 | MET14 | YKL001C | 95.9013 | 366.05 | 1.93242 | 5.00E-05 |
| 8 | MET10 | YFR030W | 52.3351 | 191.559 | 1.87194 | 5.00E-05 |
| 9 | RAD59 | YDL059C | 10.8021 | 39.2811 | 1.86252 | 0.0001 |
| 10 | MET5 | YJR137C | 29.417 | 104.471 | 1.82838 | 5.00E-05 |
| 11 | CYB5 | YNL111C | 17.1259 | 59.3361 | 1.79273 | 0.00315 |
| 12 | YPL272C | YPL272C | 15.8644 | 54.1294 | 1.77062 | 5.00E-05 |
| 13 | MET16 | YPR167C | 158.8 | 506.991 | 1.67475 | 5.00E-05 |
| 14 | SUL2 | YLR092W | 39.4596 | 125.95 | 1.6744 | 5.00E-05 |
| 15 | MET8 | YBR213W | 52.3334 | 162.844 | 1.63769 | 5.00E-05 |
| 16 | YNL276C | YNL276C | 200.409 | 608.447 | 1.60218 | 5.00E-05 |
| 17 | MET1 | YKR069W | 66.732 | 201.948 | 1.59753 | 5.00E-05 |
| 18 | GRX8 | YLR364W | 44.0763 | 132.742 | 1.59055 | 0.0001 |
| 19 | MHT1 | YLL062C | 35.8847 | 104.101 | 1.53655 | 5.00E-05 |
| 20 | CSN9 | YDR179C | 5.73674 | 16.2428 | 1.5015 | 0.03555 |
| 21 | YKL068W-A | YKL068W-A | 62.4182 | 174.715 | 1.48496 | 0.0055 |
| 22 | MET17 | YLR303W | 954.896 | 2655.33 | 1.47548 | 5.00E-05 |
| 23 | TIR3 | YIL011W | 49.6629 | 137.65 | 1.47076 | 5.00E-05 |
| 24 | MXR1 | YER042W | 172.42 | 465.489 | 1.43282 | 5.00E-05 |
| 25 | ARG5,6 | YER069W | 107.37 | 286.244 | 1.41465 | 5.00E-05 |
| 26 | ARG3 | YJL088W | 129.786 | 324.46 | 1.3219 | 5.00E-05 |
| 27 | ATF2 | YGR177C | 28.9357 | 70.5249 | 1.28528 | 5.00E-05 |
| 28 | TIR2 | YOR010C | 51.0966 | 123.044 | 1.26787 | 5.00E-05 |
| 29 | ERG3 | YLR056W | 344.877 | 830.092 | 1.26719 | 5.00E-05 |
| 30 | MET6 | YER091C | 499.117 | 1199.05 | 1.26444 | 5.00E-05 |
| 31 | FUS1 | YCL027W | 48.968 | 115.961 | 1.24373 | 5.00E-05 |
| 32 | PPT2 | YPL148C | 7.87272 | 18.5466 | 1.23622 | 0.0358 |
| 33 | SER33 | YIL074C | 162.039 | 379.922 | 1.22936 | 5.00E-05 |
| 34 | snR8 | snR8 | 85.1519 | 198.606 | 1.2218 | 0.0464 |
| 35 | CTF13 | YMR094W | 3.37262 | 7.86361 | 1.22132 | 0.0114 |
| 36 | TSA2 | YDR453C | 181.398 | 421.77 | 1.2173 | 5.00E-05 |
| 37 | YEH1 | YLL012W | 12.1723 | 28.2781 | 1.21609 | 5.00E-05 |
| 38 | SAM3 | YPL274W | 111.332 | 256.584 | 1.20456 | 5.00E-05 |
| 39 | MMP1 | YLL061W | 44.1754 | 98.7486 | 1.16052 | 5.00E-05 |
| 40 | ERG5 | YMR015C | 81.6177 | 181.806 | 1.15545 | 5.00E-05 |
| 41 | ERG25 | YGR060W | 318.553 | 690.49 | 1.11609 | 5.00E-05 |
| 42 | RMD6 | YEL072W | 40.8006 | 86.8207 | 1.08945 | 5.00E-05 |
| 43 | CRR1 | YLR213C | 3.0734 | 6.52757 | 1.08671 | 0.03515 |
| 44 | ERG11 | YHR007C | 258.804 | 542.15 | 1.06683 | 5.00E-05 |
| 45 | FMO1 | YHR176W | 19.5906 | 40.9478 | 1.06363 | 5.00E-05 |
| 46 | BIO3 | YNR058W | 7.37619 | 15.3568 | 1.05793 | 0.00115 |
| 47 | YML018C | YML018C | 97.6965 | 200.061 | 1.03406 | 5.00E-05 |
| 48 | TOD6 | YBL054W | 12.9099 | 26.3089 | 1.02707 | 5.00E-05 |
| 49 | PRM1 | YNL279W | 21.1749 | 42.687 | 1.01144 | 5.00E-05 |
| 50 | DUG3 | YNL191W | 146.601 | 295.441 | 1.01097 | 5.00E-05 |
| 51 | PAU17 | YLL025W | 16.5476 | 33.3421 | 1.01072 | 0.0467 |
| 52 | ARG8 | YOL140W | 78.6397 | 158.3 | 1.00933 | 5.00E-05 |
| 53 | MSS2 | YDL107W | 5.54309 | 11.0915 | 1.00069 | 0.0195 |
| 54 | STR3 | YGL184C | 131.04 | 260.067 | 0.988872 | 5.00E-05 |
| 55 | BUD16 | YEL029C | 21.9223 | 43.1156 | 0.975809 | 0.0005 |
| 56 | YGL194C-A | YGL194C-A | 108.687 | 213.71 | 0.975475 | 0.00735 |
| 57 | HBN1 | YCL026C-B | 13.0072 | 25.5754 | 0.975448 | 0.01855 |
| 58 | MET2 | YNL277W | 65.4826 | 127.908 | 0.965927 | 5.00E-05 |
| 59 | ADH2 | YMR303C | 9.19839 | 17.9668 | 0.965879 | 0.0064 |
| 60 | NUC1 | YJL208C | 17.7856 | 34.5598 | 0.958381 | 0.00065 |
| 61 | YMR185W | YMR185W | 7.00559 | 13.4636 | 0.942485 | 0.00025 |
| 62 | YMR141C | YMR141C | 42.0413 | 80.2406 | 0.932524 | 0.02605 |
| 63 | FAR1 | YJL157C | 62.2099 | 117.941 | 0.922846 | 5.00E-05 |
| 64 | KAR5 | YMR065W | 9.01748 | 16.8938 | 0.905693 | 0.00125 |
| 65 | MUP1 | YGR055W | 420.823 | 786.542 | 0.902309 | 5.00E-05 |
| 66 | YMR317W | YMR317W | 10.7082 | 19.9923 | 0.900727 | 5.00E-05 |
| 67 | snR84 | snR84 | 14.0538 | 26.0987 | 0.893019 | 0.03455 |
| 68 | AGP3 | YFL055W | 9.84825 | 18.2593 | 0.89069 | 0.00085 |
| 69 | TIR4 | YOR009W | 29.5026 | 54.5046 | 0.885536 | 5.00E-05 |
| 70 | RIX1 | YHR197W | 11.6183 | 21.2915 | 0.873877 | 0.0002 |
| 71 | SAM2 | YDR502C | 422.674 | 768.381 | 0.862276 | 5.00E-05 |
| 72 | PHO12 | YHR215W | 7.22859 | 13.0888 | 0.856543 | 0.00895 |
| 73 | SPP2 | YOR148C | 28.1359 | 50.8724 | 0.854471 | 0.00535 |
| 74 | BNA3 | YJL060W | 199.974 | 359.733 | 0.847113 | 5.00E-05 |
| 75 | HLR1 | YDR528W | 21.3493 | 38.3599 | 0.84541 | 0.00025 |
| 76 | VHT1 | YGR065C | 42.4964 | 75.7189 | 0.833314 | 5.00E-05 |
| 77 | AGA1 | YNR044W | 121.351 | 216.045 | 0.832145 | 5.00E-05 |
| 78 | YNL284C-B | YNL284C-B | 8.02144 | 14.2705 | 0.831106 | 0.0001 |
| 79 | IZH4 | YOL101C | 112.853 | 199.608 | 0.822724 | 5.00E-05 |
| 80 | HOP1 | YIL072W | 3.52841 | 6.23776 | 0.822009 | 0.0388 |
| 81 | DBP7 | YKR024C | 8.79893 | 15.5265 | 0.819331 | 0.00075 |
| 82 | YLR101C | YLR101C | 171.72 | 300.106 | 0.805416 | 0.00605 |
| 83 | NTC20 | YBR188C | 74.5573 | 130.124 | 0.803467 | 0.00105 |
| 84 | AUS1 | YOR011W | 10.7162 | 18.699 | 0.803159 | 5.00E-05 |
| 85 | YGR079W | YGR079W | 37.8744 | 65.6893 | 0.794436 | 0.00025 |
| 86 | GCV2 | YMR189W | 161.772 | 279.932 | 0.791118 | 5.00E-05 |
| 87 | TRF5 | YNL299W | 9.02934 | 15.5507 | 0.784288 | 0.0027 |
| 88 | FUS2 | YMR232W | 7.68191 | 13.2104 | 0.782134 | 0.00335 |
| 89 | SAM1 | YLR180W | 477.446 | 818.532 | 0.7777 | 5.00E-05 |
| 90 | RHO4 | YKR055W | 21.7842 | 37.2689 | 0.774687 | 0.00405 |
| 91 | BFR2 | YDR299W | 20.6476 | 35.1686 | 0.768309 | 0.0002 |
| 92 | MET32 | YDR253C | 86.5752 | 147.37 | 0.767418 | 0.0001 |
| 93 | CPA1 | YOR303W | 302.137 | 513.965 | 0.766466 | 5.00E-05 |
| 94 | MED11 | YMR112C | 51.1851 | 86.4098 | 0.755471 | 0.0115 |
| 95 | NOP9 | YJL010C | 8.72978 | 14.7201 | 0.753765 | 0.00375 |
| 96 | NOP8 | YOL144W | 14.2642 | 23.9614 | 0.748314 | 0.0031 |
| 97 | MNN4 | YKL201C | 34.5766 | 57.9181 | 0.744218 | 0.0001 |
| 98 | ICY2 | YPL250C | 189.326 | 317.125 | 0.744183 | 5.00E-05 |
| 99 | GGC1 | YDL198C | 260.91 | 435.347 | 0.738613 | 5.00E-05 |
| 100 | YIL080W | YIL080W | 35.2854 | 58.6548 | 0.73318 | 5.00E-05 |
| 101 | RRN11 | YML043C | 14.0301 | 23.2054 | 0.725935 | 0.003 |
| 102 | TOM7 | YNL070W | 513.172 | 848.38 | 0.725267 | 0.01385 |
| 103 | DAP1 | YPL170W | 160.474 | 264.064 | 0.718552 | 0.0001 |
| 104 | MVD1 | YNR043W | 113.912 | 187.033 | 0.715369 | 5.00E-05 |
| 105 | SCM4 | YGR049W | 237.123 | 388.907 | 0.713788 | 5.00E-05 |
| 106 | SGD1 | YLR336C | 5.93193 | 9.71776 | 0.712124 | 0.00605 |
| 107 | ARX1 | YDR101C | 15.1097 | 24.7453 | 0.711677 | 0.00175 |
| 108 | YGR201C | YGR201C | 26.2697 | 43.0008 | 0.710963 | 0.0106 |
| 109 | CYS3 | YAL012W | 651.813 | 1066.54 | 0.710403 | 5.00E-05 |
| 110 | ERG1 | YGR175C | 120.72 | 197.362 | 0.709177 | 5.00E-05 |
| 111 | YHR127W | YHR127W | 44.2541 | 72.3321 | 0.708826 | 0.0013 |
| 112 | ERG6 | YML008C | 313.522 | 512.229 | 0.708222 | 5.00E-05 |
| 113 | MMM1 | YLL006W | 24.3087 | 39.6898 | 0.707295 | 0.00065 |
| 114 | YOS1 | YER074W-A | 265.51 | 430.369 | 0.69681 | 0.00275 |
| 115 | GSH1 | YJL101C | 89.4411 | 144.802 | 0.695068 | 0.00015 |
| 116 | TGS1 | YPL157W | 14.2795 | 23.0155 | 0.68866 | 0.02085 |
| 117 | ORT1 | YOR130C | 49.9629 | 80.2616 | 0.683852 | 0.0007 |
| 118 | NFT1 | YKR103W | 3.71393 | 5.96186 | 0.682815 | 0.01265 |
| 119 | YHR112C | YHR112C | 93.8868 | 150.58 | 0.681535 | 5.00E-05 |
| 120 | ATC1 | YDR184C | 44.4166 | 71.1339 | 0.679438 | 0.002 |
| 121 | YJR149W | YJR149W | 16.646 | 26.5833 | 0.675343 | 0.00645 |
| 122 | SNU66 | YOR308C | 7.36733 | 11.7628 | 0.675015 | 0.02125 |
| 123 | MET30 | YIL046W | 47.2607 | 75.3842 | 0.673622 | 0.0001 |
| 124 | YNL089C | YNL089C | 31.7285 | 50.5378 | 0.671586 | 0.03845 |
| 125 | RBG2 | YGR173W | 17.9223 | 28.5314 | 0.670795 | 0.0076 |
| 126 | ENP1 | YBR247C | 20.3255 | 32.3551 | 0.670699 | 0.0026 |
| 127 | YCT1 | YLL055W | 25.6555 | 40.7122 | 0.666193 | 0.00045 |
| 128 | TSR2 | YLR435W | 25.8587 | 40.9456 | 0.663059 | 0.02325 |
| 129 | DHR2 | YKL078W | 4.13688 | 6.52987 | 0.658512 | 0.04705 |
| 130 | RBG1 | YAL036C | 32.9258 | 51.8969 | 0.656431 | 0.0013 |
| 131 | ERG2 | YMR202W | 340.476 | 536.411 | 0.655784 | 0.0001 |
| 132 | FIG2 | YCR089W | 19.162 | 30.1555 | 0.654176 | 0.0001 |
| 133 | RIX7 | YLL034C | 11.543 | 18.1614 | 0.653855 | 0.0021 |
| 134 | GPB1 | YOR371C | 9.57866 | 15.0483 | 0.651705 | 0.00315 |
| 135 | snR17a | snR17a | 300.097 | 471.186 | 0.650869 | 0.0029 |
| 136 | PZF1 | YPR186C | 10.6195 | 16.6702 | 0.650562 | 0.02345 |
| 137 | DUG2 | YBR281C | 38.4936 | 60.249 | 0.64632 | 0.0001 |
| 138 | RSA4 | YCR072C | 12.8239 | 20.0156 | 0.642293 | 0.0064 |
| 139 | URA10 | YMR271C | 42.6459 | 66.4823 | 0.640562 | 0.00605 |
| 140 | SSF1 | YHR066W | 39.2092 | 61.1127 | 0.640282 | 0.0007 |
| 141 | SHM2 | YLR058C | 591.546 | 920.395 | 0.637763 | 0.00035 |
| 142 | YDL063C | YDL063C | 15.4408 | 24.0149 | 0.637182 | 0.0033 |
| 143 | LCP5 | YER127W | 21.759 | 33.8079 | 0.635752 | 0.007 |
| 144 | PUF6 | YDR496C | 53.6364 | 83.2787 | 0.634734 | 0.0004 |
| 145 | NOG2 | YNR053C | 34.683 | 53.742 | 0.631823 | 0.0015 |
| 146 | NCS2 | YNL119W | 22.2597 | 34.2954 | 0.623581 | 0.0031 |
| 147 | AIR1 | YIL079C | 38.4675 | 59.2217 | 0.622486 | 0.00205 |
| 148 | YAR009C | YAR009C | 8.50193 | 13.0868 | 0.622248 | 0.00375 |
| 149 | MET22 | YOL064C | 143.449 | 220.62 | 0.621028 | 0.00015 |
| 150 | ATM1 | YMR301C | 47.1092 | 72.3216 | 0.618418 | 0.0004 |
| 151 | SLX9 | YGR081C | 24.1615 | 37.032 | 0.616059 | 0.0378 |
| 152 | MET13 | YGL125W | 93.7614 | 143.612 | 0.615109 | 0.0003 |
| 153 | NRG2 | YBR066C | 107.805 | 164.862 | 0.612835 | 0.00095 |
| 154 | PCH2 | YBR186W | 8.86771 | 13.5392 | 0.610511 | 0.0239 |
| 155 | ERG26 | YGL001C | 94.5631 | 144.205 | 0.608768 | 0.00015 |
| 156 | SYS1 | YJL004C | 26.3751 | 40.1451 | 0.606046 | 0.03905 |
| 157 | CMS1 | YLR003C | 38.8643 | 59.1537 | 0.606023 | 0.0066 |
| 158 | PCL2 | YDL127W | 54.4266 | 82.6929 | 0.603452 | 0.00285 |
| 159 | RRS1 | YOR294W | 40.4457 | 61.2663 | 0.599107 | 0.0138 |
| 160 | SMC3 | YJL074C | 5.80857 | 8.78387 | 0.596673 | 0.01385 |
| 161 | YDL050C | YDL050C | 185.623 | 280.425 | 0.595241 | 0.01835 |
| 162 | ENP2 | YGR145W | 13.4303 | 20.2765 | 0.594314 | 0.00705 |
| 163 | MIT1 | YEL007W | 117.79 | 78.5108 | -0.58525 | 0.0006 |
| 164 | CIT2 | YCR005C | 491.632 | 327.078 | -0.58794 | 0.00075 |
| 165 | TPO4 | YOR273C | 125.893 | 83.6622 | -0.58955 | 0.00045 |
| 166 | MED2 | YDL005C | 110.739 | 73.5794 | -0.58979 | 0.00055 |
| 167 | VID24 | YBR105C | 119.38 | 79.3039 | -0.5901 | 0.0009 |
| 168 | PUS6 | YGR169C | 35.0654 | 23.2322 | -0.59393 | 0.0071 |
| 169 | ERV14 | YGL054C | 838.626 | 555.596 | -0.59399 | 0.0005 |
| 170 | MSN4 | YKL062W | 121.318 | 80.37 | -0.59406 | 0.0002 |
| 171 | ALF1 | YNL148C | 31.203 | 20.671 | -0.59408 | 0.0403 |
| 172 | HXT4 | YHR092C | 449.316 | 297.301 | -0.59581 | 0.00035 |
| 173 | RPS20 | YHL015W | 6287.44 | 4152.26 | -0.59858 | 0.00095 |
| 174 | PEX11 | YOL147C | 87.0021 | 57.403 | -0.59992 | 0.0032 |
| 175 | DIF1 | YLR437C | 119.104 | 78.151 | -0.60788 | 0.01565 |
| 176 | BNA7 | YDR428C | 30.2978 | 19.8655 | -0.60895 | 0.03315 |
| 177 | SKS1 | YPL026C | 35.8218 | 23.4511 | -0.61118 | 0.00345 |
| 178 | SSE2 | YBR169C | 120.848 | 78.9803 | -0.61363 | 0.0001 |
| 179 | YNR034W-A | YNR034W-A | 716.415 | 467.855 | -0.61473 | 0.0008 |
| 180 | UME6 | YDR207C | 39.6623 | 25.8734 | -0.6163 | 0.0007 |
| 181 | RFM1 | YOR279C | 35.0585 | 22.8596 | -0.61696 | 0.01055 |
| 182 | YBR085C-A | YBR085C-A | 257.544 | 167.727 | -0.6187 | 0.0235 |
| 183 | RTN2 | YDL204W | 267.874 | 174.389 | -0.61924 | 0.00015 |
| 184 | MIG1 | YGL035C | 130.733 | 84.9017 | -0.62276 | 0.00015 |
| 185 | TES1 | YJR019C | 64.9823 | 42.1201 | -0.62554 | 0.00415 |
| 186 | YER163C | YER163C | 66.3071 | 42.779 | -0.63226 | 0.00525 |
| 187 | RTC2 | YBR147W | 241.378 | 155.712 | -0.63241 | 5.00E-05 |
| 188 | YCR087C-A | YCR087C-A | 117.27 | 75.5908 | -0.63355 | 0.0082 |
| 189 | LYS1 | YIR034C | 1508.37 | 970.869 | -0.63564 | 0.00055 |
| 190 | ISU1 | YPL135W | 169.383 | 108.793 | -0.63871 | 0.0018 |
| 191 | ICS2 | YBR157C | 90.6845 | 58.1578 | -0.64089 | 0.00145 |
| 192 | ICY1 | YMR195W | 1740.37 | 1114.77 | -0.64265 | 5.00E-05 |
| 193 | SGA1 | YIL099W | 9.6075 | 6.14375 | -0.64504 | 0.04395 |
| 194 | UBP9 | YER098W | 19.4022 | 12.4038 | -0.64545 | 0.0032 |
| 195 | BCK2 | YER167W | 41.2557 | 26.3139 | -0.64877 | 0.00035 |
| 196 | TSL1 | YML100W | 216.148 | 137.594 | -0.6516 | 5.00E-05 |
| 197 | IGD1 | YFR017C | 250.115 | 159.135 | -0.65234 | 0.0005 |
| 198 | PMP3 | YDR276C | 2975.26 | 1885.44 | -0.65812 | 0.00275 |
| 199 | YHL030W-A | YHL030W-A | 129.251 | 81.5649 | -0.66415 | 0.01305 |
| 200 | RHO5 | YNL180C | 279.571 | 176.348 | -0.66479 | 5.00E-05 |
| 201 | RTS3 | YGR161C | 275.99 | 172.911 | -0.67458 | 5.00E-05 |
| 202 | THI2 | YBR240C | 10.6198 | 6.62896 | -0.67991 | 0.04715 |
| 203 | DIP5 | YPL265W | 227.702 | 141.414 | -0.68722 | 5.00E-05 |
| 204 | GUT2 | YIL155C | 61.6887 | 38.2807 | -0.68839 | 0.0001 |
| 205 | IRC7 | YFR055W | 88.6786 | 54.7982 | -0.69446 | 0.0002 |
| 206 | YDR391C | YDR391C | 163.138 | 100.603 | -0.69741 | 0.0002 |
| 207 | NCE103 | YNL036W | 450.401 | 277.664 | -0.69787 | 5.00E-05 |
| 208 | BAG7 | YOR134W | 20.325 | 12.528 | -0.6981 | 0.0107 |
| 209 | YPS6 | YIR039C | 20.8698 | 12.8589 | -0.69865 | 0.00345 |
| 210 | GAC1 | YOR178C | 36.4671 | 22.2123 | -0.71524 | 5.00E-05 |
| 211 | MGA1 | YGR249W | 54.3946 | 32.8672 | -0.72681 | 0.0001 |
| 212 | NDE1 | YMR145C | 242.312 | 146.343 | -0.72751 | 5.00E-05 |
| 213 | COS111 | YBR203W | 18.6418 | 11.2304 | -0.73113 | 0.0006 |
| 214 | FMP45 | YDL222C | 26.8863 | 16.1952 | -0.73131 | 0.011 |
| 215 | MTL1 | YGR023W | 45.9756 | 27.6705 | -0.73252 | 0.0001 |
| 216 | GIP2 | YER054C | 46.9683 | 28.2471 | -0.73359 | 0.0002 |
| 217 | KNH1 | YDL049C | 35.2513 | 21.1833 | -0.73475 | 0.008 |
| 218 | YNR014W | YNR014W | 232.484 | 138.744 | -0.7447 | 5.00E-05 |
| 219 | YFL054C | YFL054C | 22.6338 | 13.4427 | -0.75165 | 0.00075 |
| 220 | LYS12 | YIL094C | 629.16 | 372.21 | -0.75731 | 5.00E-05 |
| 221 | COX14 | YML129C | 300.917 | 176.712 | -0.76796 | 0.0298 |
| 222 | YAP6 | YDR259C | 17.5625 | 10.2874 | -0.77162 | 0.01435 |
| 223 | SIP4 | YJL089W | 7.28991 | 4.24039 | -0.78171 | 0.0122 |
| 224 | SHU2 | YDR078C | 24.076 | 13.9922 | -0.78297 | 0.03025 |
| 225 | YDR461C-A | YDR461C-A | 452.774 | 262.793 | -0.78487 | 0.0015 |
| 226 | MAL11 | YGR289C | 11.9436 | 6.90852 | -0.78979 | 0.009 |
| 227 | RGM1 | YMR182C | 79.296 | 45.5734 | -0.79906 | 0.0004 |
| 228 | PRM10 | YJL108C | 27.2584 | 15.5647 | -0.80842 | 0.0047 |
| 229 | ADR1 | YDR216W | 11.299 | 6.4109 | -0.81759 | 0.0002 |
| 230 | YRO2 | YBR054W | 806.196 | 455.877 | -0.82249 | 5.00E-05 |
| 231 | SDP1 | YIL113W | 67.3338 | 37.1217 | -0.85907 | 0.0009 |
| 232 | ADH1 | YOL086C | 3681.23 | 2023.72 | -0.86318 | 0.001 |
| 233 | AAC1 | YMR056C | 16.2271 | 8.85034 | -0.8746 | 0.01695 |
| 234 | QRI5 | YLR204W | 124.642 | 67.7468 | -0.87956 | 0.005 |
| 235 | FIT2 | YOR382W | 357.81 | 193.941 | -0.88357 | 5.00E-05 |
| 236 | YER064C | YER064C | 97.5624 | 52.6552 | -0.88975 | 5.00E-05 |
| 237 | PHM7 | YOL084W | 7.44508 | 3.98894 | -0.90028 | 0.0041 |
| 238 | YDL218W | YDL218W | 12.7805 | 6.81185 | -0.90782 | 0.02745 |
| 239 | YPL277C | YPL277C | 7.72853 | 4.08452 | -0.92003 | 0.02575 |
| 240 | LYS9 | YNR050C | 1396.38 | 720.416 | -0.95479 | 5.00E-05 |
| 241 | DUR1,2 | YBR208C | 49.2058 | 25.1317 | -0.96932 | 5.00E-05 |
| 242 | CAR2 | YLR438W | 71.7443 | 36.0621 | -0.99238 | 5.00E-05 |
| 243 | BDH2 | YAL061W | 81.9014 | 40.8422 | -1.00383 | 5.00E-05 |
| 244 | QCR9 | YGR183C | 216.952 | 108.1 | -1.005 | 0.03465 |
| 245 | CTT1 | YGR088W | 166.662 | 80.7437 | -1.0455 | 5.00E-05 |
| 246 | FMP43 | YGR243W | 106.332 | 50.6233 | -1.0707 | 5.00E-05 |
| 247 | ECM8 | YBR076W | 6.94923 | 3.28125 | -1.08261 | 0.04655 |
| 248 | YJR115W | YJR115W | 183.402 | 86.5745 | -1.083 | 5.00E-05 |
| 249 | FMP48 | YGR052W | 145.291 | 68.4368 | -1.0861 | 5.00E-05 |
| 250 | DDR2 | YOL052C-A | 272.373 | 125.574 | -1.11704 | 0.03815 |
| 251 | XBP1 | YIL101C | 52.0234 | 23.7962 | -1.12843 | 5.00E-05 |
| 252 | HXT2 | YMR011W | 106.174 | 48.422 | -1.13269 | 5.00E-05 |
| 253 | HAP4 | YKL109W | 149.822 | 67.5646 | -1.14891 | 5.00E-05 |
| 254 | ALP1 | YNL270C | 15.3889 | 6.92023 | -1.153 | 0.00015 |
| 255 | SLZ1 | YNL196C | 8.65093 | 3.88543 | -1.15478 | 0.0393 |
| 256 | SPG3 | YDR504C | 30.8542 | 13.7859 | -1.16227 | 0.0398 |
| 257 | PUT4 | YOR348C | 10.7133 | 4.71215 | -1.18495 | 0.00085 |
| 258 | HSP12 | YFL014W | 721.574 | 310.124 | -1.2183 | 5.00E-05 |
| 259 | CAR1 | YPL111W | 201.974 | 83.6247 | -1.27217 | 5.00E-05 |
| 260 | ISF1 | YMR081C | 120.788 | 48.9856 | -1.30205 | 5.00E-05 |
| 261 | ATG16 | YMR159C | 19.2369 | 7.7009 | -1.32078 | 0.0421 |
| 262 | DAK2 | YFL053W | 6.98805 | 2.70249 | -1.3706 | 0.0053 |
| 263 | CYC7 | YEL039C | 92.6801 | 35.0863 | -1.40135 | 0.0015 |
| 264 | YKR075C | YKR075C | 74.1587 | 26.701 | -1.47372 | 5.00E-05 |
| 265 | HXT12 | YIL170W | 6.87955 | 2.42161 | -1.50635 | 0.01005 |
| 266 | ECM13 | YBL043W | 24.3414 | 8.1611 | -1.57657 | 0.00075 |
| 267 | CIN5 | YOR028C | 118.924 | 35.3114 | -1.75183 | 5.00E-05 |
| 268 | ASG7 | YJL170C | 11.5686 | 3.2186 | -1.84571 | 0.0247 |
| 269 | RCK1 | YGL158W | 14.7116 | 3.61906 | -2.02326 | 0.00015 |
| 270 | CRC1 | YOR100C | 6.78777 | 1.40672 | -2.2706 | 0.02475 |
